# Volatile Organic Compounds (VOCs) in Conventional and High Performance School Buildings in the U.S.

**DOI:** 10.3390/ijerph14010100

**Published:** 2017-01-21

**Authors:** Lexuan Zhong, Feng-Chiao Su, Stuart Batterman

**Affiliations:** Environmental Health Sciences, School of Public Health, University of Michigan, Ann Arbor, MI 48109, USA; lexuanz@umich.edu (L.Z.); joefcsu@umich.edu (F.-C.S.)

**Keywords:** volatile organic compounds (VOCs), formaldehyde, schools, indoor/outdoor (I/O) ratio, air exchange rate (AER), high performance

## Abstract

Exposure to volatile organic compounds (VOCs) has been an indoor environmental quality (IEQ) concern in schools and other buildings for many years. Newer designs, construction practices and building materials for “green” buildings and the use of “environmentally friendly” products have the promise of lowering chemical exposure. This study examines VOCs and IEQ parameters in 144 classrooms in 37 conventional and high performance elementary schools in the U.S. with the objectives of providing a comprehensive analysis and updating the literature. Tested schools were built or renovated in the past 15 years, and included comparable numbers of conventional, Energy Star, and Leadership in Energy and Environmental Design (LEED)-certified buildings. Indoor and outdoor VOC samples were collected and analyzed by thermal desorption, gas chromatography and mass spectroscopy for 94 compounds. Aromatics, alkanes and terpenes were the major compound groups detected. Most VOCs had mean concentrations below 5 µg/m^3^, and most indoor/outdoor concentration ratios ranged from one to 10. For 16 VOCs, the within-school variance of concentrations exceeded that between schools and, overall, no major differences in VOC concentrations were found between conventional and high performance buildings. While VOC concentrations have declined from levels measured in earlier decades, opportunities remain to improve indoor air quality (IAQ) by limiting emissions from building-related sources and by increasing ventilation rates.

## 1. Introduction

Indoor air quality (IAQ) and, more broadly, indoor environmental quality (IEQ) have received considerable attention from the public as well as from practitioners and researchers. After homes, schools are the most important indoor environment for children, who spend over 1000 hours each year at school. Unfortunately, school environments are often deficient, which may adversely affect student performance and attendance [1,2,3,4,5,6]. Moreover, students represent a potentially vulnerable population, one that may be especially susceptible to pollutant exposure. Even low levels of pollutants such as carbon dioxide (CO_2_), volatile organic compounds (VOCs), and particulate matter (PM_2.5_ and PM_10_) have been associated with the development of respiratory and other adverse health outcomes in children [7,8].

A modest number of studies have examined IEQ and particularly VOCs in schools. VOCs encompass a very wide range of chemicals, and many may cause acute or chronic health effects. These chemicals arise from both indoor and outdoor sources; thus, indoor and outdoor VOC concentrations are often correlated and sometimes vary seasonally [9,10,11]. The VOCs most commonly measured include benzene, toluene, xylene, ethylbenzene, α-pinene, and d-limonene [9,10,11,12,13,14,15]. Several studies have focused on characterizing selected IAQ parameters and evaluating the impacts of pollutant exposure on children’s health [16,17], but relatively few have provided comprehensive measurements of VOCs, the emphasis of the present paper. Table S1 summarizes the literature on VOCs.

The building industry has evolved significantly over the past several decades with the introduction and growing use of “green” building certification systems, such as the U.S. Green Building Council’s Leadership in Energy and Environmental Design (LEED) program, launched in 2000 [18]. Several school districts have begun to construct new schools and renovate old schools to attain LEED or other certifications with the intentions of reducing energy consumption and improving the environment and health of employees and students. Such “high performance” buildings are designed to improve IEQ and, potentially, to reduce distraction and positively affect learning ability, test scores, staff/student attendance, and employee satisfaction [19,20]. However, green building rating schemes give IAQ considerations an average of only 7.5% of the overall score [21]. In addition, due to concerns of the (high) energy consumption of traditional ventilation, ASHRAE (American Society of Heating, Refrigerating and Air-Conditioning Engineers) Standard 62.1 allows low ventilation rates if contaminant exposures are reduced by alternative means, e.g., air filtration and cleaning systems [22,23]. Such systems can also gain LEED credits.

In parallel with the green building rating systems, the formulation of many materials and the practices used in buildings have evolved to be more “environmentally friendly”. Low VOC-emitting building materials and consumer products have reduced VOC concentrations, as demonstrated in Korea [24], Germany [25], Taiwan [26], and elsewhere. LEED and other certification systems also provide credits for using low-emitting materials. Emerging air cleaning techniques, such as ultraviolet photocatalytic oxidation [27], have the potential to abate indoor VOCs while reducing the ventilation of air.

In addition, laws and policies pertaining to ambient air quality have dramatically lowered concentrations of many outdoor pollutants over the past several decades. This also can improve the IAQ since the rudimentary air cleaning systems in most buildings, e.g., HVAC (Heating, ventilation and air conditioning) filters that reduce only particulate matter concentrations, allow VOCs in outdoor air to enter the occupied portion of buildings.

This paper reports on indoor and outdoor VOC measurements in 144 classrooms at 37 recently constructed or renovated schools in the U.S. Midwest. This project aims to characterize recent VOC levels in a wide cross-section of schools and contrast results for conventional and high performance school buildings.

## 2. Materials and Methods

### 2.1. Selection and Characteristics of School Buildings

Thirty-seven elementary and K-8 schools built or renovated within the past 15 years were selected in the U.S. states of Ohio, Illinois, Michigan, and Indiana. Of these, 24 were newly constructed and 13 had complete renovations. Of the 27 “high performance” schools, 12 (44%) were LEED certified or LEED designed (constructed from 2003 to 2012), and 15 (56%) met the U.S. Environmental Protection Agency (EPA) Energy Star (ES) criteria (constructed from 2002 to 2010). The remaining 10 schools (27%) had conventional designs (built or renovated from 2001 to 2011). All of them were permanent buildings, i.e., no portable classrooms were included in the sample. Up to three schools each week were visited from 20 October 2015 through 30 March 2016 for sampling, walk-though inspections of the building, study classrooms, HVAC systems and school environs, and for other assessments.

The building typologies differed greatly. There were 14 two-story buildings, six three-floor buildings (including split level), and 17 single-story slab-on-grade buildings. All buildings were mechanically ventilated: 26 relied on central air handling units (AHUs); four used only classroom unit ventilators (UVs); two used a mix of AHU and UVs; and five used geothermal water-to-air pumps for both heating and cooling and central AHUs. HVAC filters were diverse: 15 schools used 2 in pleated MERV-8 (Minimum efficiency reporting value) filters; 17 schools used 2 in higher MERV filters; and five schools with classroom UVs and geothermal heat pumps used pleated or polyring panel or flat panel filters with several depths and generally lower MERV ratings. Several buildings were near freeways (13%) or other highways (8%), and 32% were close to industrial areas.

Within each school, four classrooms were selected for assessment, based on the principal’s advice and the teacher’s willingness. A total of 144 rooms were sampled, which included 139 general classrooms, two music rooms, one art room, and two resource rooms. Table S2 summarizes school building characterization. These rooms served mostly kindergarten to 5th grade students, although a few prekindergarten and 7th and 8th grade classrooms were included. A walk-through inspection was performed in each school and room studied, and teachers were queried regarding the number of students present in their classroom, classroom activities, and IEQ perceptions. Most classrooms had exterior walls with windows (85%). A subset had doors to the outside (18%), passage doors to an adjoining restroom (30%), and most had sinks within the classroom (86%). Teachers could open windows in most (85%) rooms, but 13% of teachers reported that the windows were never opened. A few classrooms (14%) were near bus idling areas, and an additional 13% were near parking lots or other areas where vehicles may idle.

All teachers in the tested schools were invited to participate in an online health and IEQ perception survey. A total of 318 teachers provided complete responses, of which 89 teachers also had IEQ measurements conducted within their classroom.

### 2.2. Air Quality Sampling

Indoor samplers were set up in representative locations, e.g., on a teacher’s desk or a spare student’s table, approximately 0.6 m above the floor, and away from windows, doors, supply or return diffusers, potential emission sources, and out of direct sunlight. An identical sampler was deployed outdoors, either on the school’s roof or the ground. At each school, samples at these five sites (four classrooms and one outdoor site, and sometimes a sixth cafeteria site) were obtained simultaneously over two mid-week school days.

Monitored parameters included integrated samples of VOCs and formaldehyde, continuous measurements of temperature, relative humidity (RH), CO_2_, particle number, and noise. VOCs were collected using diffusion (passive) samplers (see below). The short, one- to two-day sampling period in phase 1 of this project was designed to provide rapid screening of VOC levels among a cross-section of 37 recently constructed elementary school buildings. In phase 2 of the study, we will obtained repeated observations at a subset of schools and extend the sampling period. Formaldehyde was monitored using an electrochemical analyzer (GrayWolf FM-801, GrayWolf Sensing Solutions, Shelton, CT, USA; detection limit of 6 µg/m^3^) for a minimum of 30 min in each room.

### 2.3. VOC Sampling and Analysis

VOC samplers used 10 cm stainless sorbent tubes packed with 160 mg 60/80 mesh Tenax-GR with a 0.5 cm diffusion gap. Before sampling, tubes were conditioned at 325 °C for 6 h with a 30 mL/min flow of high purity N_2_, then capped, wrapped in baked aluminum foil, placed in a glass jar with an activated carbon pack, and refrigerated. During sampling, the cap was removed and the tube was placed in a stand allowing free air circulation. The sampling uptake rate was calculated using a diffusion model that depends on temperature, tube configuration, and the diffusion coefficient of each target compound [28]. After sampling, the cap was replaced, and the tube stored as described earlier.

In the laboratory, collected analytes were analyzed using an automated thermal desorber (ATD) system (Model 2000, Scientific Instrument Services, Ringoes, NJ, USA), and gas chromatograph/mass spectrometer (GC/MS, Model 6890/5973, Agilent Technologies, Santa Clara, CA, USA). The GC used a capillary column (DB-VRX, 60 m, 0.25 mm, 1.40 µm, Agilent Technologies, Santa Clara, CA, USA) and ChemStation software (G1701BA, Agilent Technologies, Santa Clara, CA, USA). The cryotrap cooling, heating and desorption temperatures were −140, 250 and 200 °C, respectively, and the injector, detector, MS quadruple, and MS source temperatures were 250, 250, 150, and 230 °C, respectively. The GC oven temperature was initially 45 °C for 10 min, increased at 8 °C/min to 140 °C, held for 10 min, then increased at 30 °C/min to 225 °C, and held for 13 min. The MS was operated in scan mode from 29–270 AMU (Atomic mass unit). Internal standards and other operating conditions and performance evaluations are described elsewhere [29].

Calibration used a 94-component mixture (EPA 524.2 Fortification Solution, EPA 524.2 Rev 4 Update Ketones Mix, EPA 502/524 Volatiles Organic Calibration Mix A, EPA 524.2 Rev 4 Update Mix, alkanes, and terpenes from Sigma Aldrich, Santa Clara, CA, USA) with loadings of 1, 3, 10, 30, and 100 ng. Prior to the analysis of samples and blanks, daily quality assurance (QA) checks were performed that included analyzing a 10 ng QA sample with acceptance criterion of 30%. Method detection limits (MDL), determined as the standard deviation of seven replicate low concentration injections (0.4 ng) multiplied by 3.14 (the Student’s *t*-value at the 99% confidence level), ranged from 0.06 to 0.25 µg/m^3^ for most target VOCs (Table S3). Non-detects were set to one-half of the MDL. Duplicate samples were collected and analyzed at three schools; and field blanks were employed at every school to access possible contamination during transport, storage, and handling.

### 2.4. Air Exchange Rates

The air exchange rate (AER) for each classroom was calculated using measured CO_2_ concentrations, room dimensions, occupancy data, and a time-dependent numerical mass balance model that simulated 15 min CO_2_ concentrations over the study period [30]. Separate AERs were estimated for occupied (school day) and unoccupied (evening and early morning) periods. In most cases, the simulations showed excellent agreement with CO_2_ measurements, and AERs were robust and stable, i.e., not sensitive to the specific period or day examined. In all cases, AERs were very low during unoccupied periods when the building’s HVAC system was shutdown.

### 2.5. Data Analysis

The detection frequency (DF), defined as the fraction of measurements above the MDL, was calculated for each VOC. Considering the short sampling duration (one to two days in this study) and relatively low uptake rate of the passive sampler, DFs were anticipated to be somewhat low, despite the sensitivity of the ATD/GC/MS system. The analysis considered only compounds with DFs above 15% (Table S4). Concentrations within several VOC groups (aromatics, aliphatics, terpenes) were summed, and the total target VOC (TTVOC) concentration was calculated as a summary measure.

Distributions of concentrations were visualized using lognormal probability plots. Correlations between VOC concentrations and IEQ measurements were calculated using Spearman rank correlation coefficients. Indoor to outdoor (I/O) ratios, a simple technique to examine the relationship between indoor and outdoor concentrations, were calculated for each VOC.

Within- and between-school variances of indoor VOC concentrations were apportioned and tested using random effects models and a completely nested design. To obtain the balanced data necessary for this analysis, a few missing VOC measurements were replaced by average values from the other classrooms in the same school (four schools were missing measurements of one classroom).

Differences in concentrations among school types (conventional, EnergyStar, LEED), locations (no emission sources, near road, and near industry), and seasons (fall = October–December 2015, winter = January–February 2016, and spring = March 2016) were examined using one-way ANOVA and Kruskal Wallis (K-W) tests. Outdoor and average indoor VOCs were compared using paired t and Wilcoxon signed rank sum tests. Associations between VOCs and classroom characteristics (e.g., floor/furniture materials, art/science/cleaning supplies/plants/animals, near outside emission sources) were examined using log-transformed concentrations and linear regression models.

Statistical analyses and model fitting were performed using Excel (Microsoft 2013, Seattle, WA, USA), SAS 9.4 (SAS Institute, Cary, NC, USA) and SPSS Statistics v. 23 (SPSS Corporation, Chicago, IL, USA).

## 3. Results

### 3.1. Indoor and Outdoor VOC Concentrations

The outdoor air contained 10 target VOCs with DFs exceeding 15% (Table 1). Of these, the most prevalent were aromatic compounds, e.g., toluene, benzene, *p,m*-xylene and 1,2,4-trimethylbenzene (1,2,4-TMB), and the VOCs with the highest median concentrations were n-hexane (C_6_, 2.4 µg/m^3^), methyl cyclohexane (MCH, 1.3 µg/m^3^), and methylene chloride (DCM, 0.8 µg/m^3^). While there are many sources of these VOCs, hexane and MCH are constituents of gasoline and other fuels, and DCM is a common component in paint and cleaning products and a globally distributed VOC. Most other VOCs had low concentrations, e.g., six of the 10 VOCs had median concentrations below 1 µg/m^3^.

Indoors, the 15% DF criterion was exceeded by 24 different VOCs, which included aromatics, alkanes, terpenes, esters, ethers, ketones and chlorinated species (Table 1). Concentrations spanned a large range, especially the terpenes. Benzene, toluene, *p,m*-xylene, C_6_, *n*-tetradecane (C_14_), and *d*-limonene were found in most (>80%) classrooms. Formaldehyde, MCH, DCM, and *d*-limonene had the highest median concentrations (0.8 to 6.0 µg/m^3^). Seven alkanes were found indoors (DF > 15%), but not outdoors (excluding C_6_). C_6_ and C_14_ had the highest median concentrations, but the levels of most alkanes fell below 2.2 µg/m^3^. Aromatic VOCs had mostly low concentrations, below 1 µg/m^3^, as in the outdoor samples. VOC compositions in the classrooms suggested the contribution of outdoor sources.

No VOC was detected in blank tubes, and duplicate precision averaged 20%. This performance met our quality assurance (QA) targets, despite the short sampling period and the sampling tube’s small diffusion gap.

**Figure 1:**
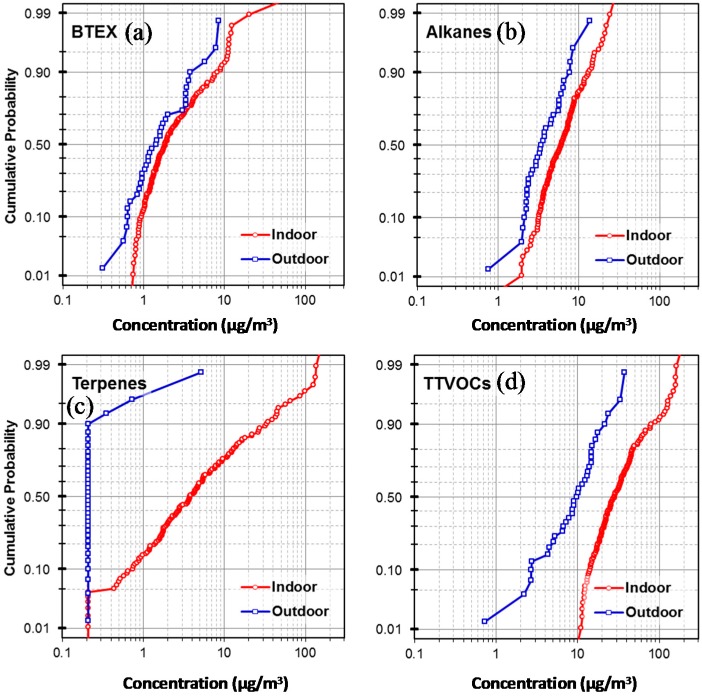
Probability plots of (**a**) BTEX (Benzene, toluene, ethylbenzene and xylene), (**b**) alkanes (C_6_–C_7_, C_11_–C_16_), (**c**) terpenes (α-pinene and *d*-limonene), and (**d**) TTVOCs (total target VOCs) in 144 school rooms and 35 outdoor locations.

Concentrations in the three compound groups were strongly (often r > 0.5) correlated across classrooms in the study, i.e., compounds within the BTEX (Benzene, toluene, ethylbenzene and xylene) group (benzene, toluene, ethylbenzene, and xylene); the alkane group (C_6_, C_7_, C_11_–C_16_); and the terpenoid group (α-pinene and d-limonene). (The Spearman rank correlation coefficients are shown in Tables S5–S7.) These groups often arise from similar sources, e.g., BTEX are fuel- and solvent-related; alkanes are components of lubricants and many other products; and terpenes are used as fragrances and cleaners. Hence, concentrations in these groups were summed for some analyses. Unsurprisingly, concentrations in the BTEX, alkane and terpene groups (along with formaldehyde) were significantly correlated with TTVOC (0.4 ≤ r ≤ 0.8). Benzene had the lowest correlation coefficient with TTVOC (r = 0.22, *p* = 0.01). Probability plots of individual VOCs and compound group concentrations suggest that alkanes, terpenes and TTVOC concentrations had approximately lognormal distributions and several possible outliers (Figure 1).

These results reflect the many potential emission sources of VOCs found in schools. The walk-through surveys noted, for example, art supplies in 15% of classrooms (e.g., acrylic/tempera paints, spray paints, and permanent markers containing aromatic- and alkane-containing solvents and adhesives), cleaning supplies in 37% of classrooms (e.g., α-pinene, *d*-limonene), and many styles of “air fresheners” in 25% of classrooms (e.g., candles, sprays, plug-in fresheners, and aromatic oil-warmers, many also likely to contain α-pinene and *d*-limonene). VOC levels in the two music rooms, the art room, and the two resource rooms sampled were comparable with the other general classrooms, except that tetrahydrofuran (0.95 µg/m^3^) was found in only the art room. A classroom in a conventional school with a plug-in air freshener had the highest limonene concentration (159 µg/m^3^ in S8C3, S stands for school ID and C stands for room ID). Two classrooms in the same EnergyStar (ES) school had the highest levels of MCH (53 µg/m^3^ in S20C1) and 1,2,4-TMB (4.2 µg/m^3^ in S20C2), and two other ES schools had the highest levels of hexane (27 µg/m^3^ in S26C3) and toluene (84 µg/m^3^ in S3C4). A LEED school had the highest α-pinene level (56 µg/m^3^ in S23C1).

### 3.2. Indoor/Outdoor (I/O) Relationship

**Figure 2:**
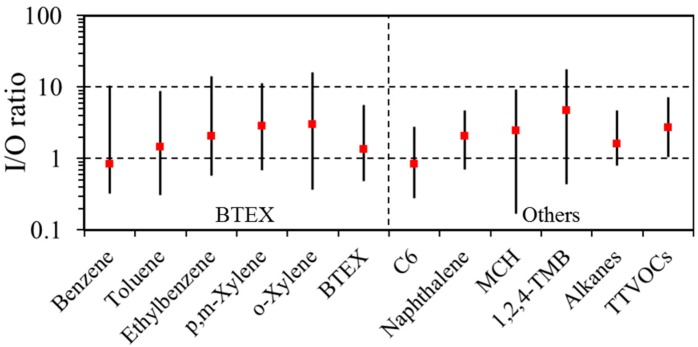
Indoor/outdoor (I/O) concentration ratios at 10th, 50th (red square) and 90th percentiles.

Figure 2 shows I/O ratios at the 10th, 50th and 90th percentile levels by compound group. In general: I/O ratios near one indicate compounds that have primarily outdoor sources (e.g., typically benzene from gasoline and other vehicle-related emissions); ratios in the one to 10 range suggest VOCs from both indoor and outdoor sources (e.g., toluene from outdoor gasoline-related emissions and indoor paints, adhesives and solvents); and I/O ratios above 10 indicate primarily or exclusively indoor sources (e.g., *d-*limonene and naphthalene from fragrances and deodorizers). Median I/O ratios were near or below one for benzene and C_6_, and between one to 10 for most of the VOCs and VOC groups. An I/O ratio above 10 would have been calculated for *d*-limonene, a common component of cleaning products used in the schools; however, ratios for this compound were not calculated given its low DF in the outdoor samples. I/O ratios reflect multiple effects beyond the relative strength of indoor and outdoor sources, e.g., ratios are affected by the air exchange rate (AER), physico-chemical reactions, source-sink effects, and detection limits.

The I/O analysis is extended in Table 1, which shows that indoor concentrations were statistically higher than outdoor levels for many VOCs (e.g., ethyl acetate, MIBK (Methyl isobutyl ketone), C_7_, C_11_C_16_, alkanes, BTEX, *p*-DCB (1,4-Dichlorobenzene), terpenes, and TTVOCs). Five VOCs (benzene, C6, MCH, DCM and chloroform) did not show differences, based on *p*-values of paired sample *t*-tests and Wilcoxon signed-rank tests. In general, the statistical tests, I/O ratios, and log probability plots gave consistent results.

## 4. Discussion

### 4.1. Effect of Indoor Environment Parameters

Indoor VOC concentrations were positively correlated with several indoor parameters, including relative humidity (RH; especially formaldehyde; r = 0.50), average CO_2_ levels, and classroom occupancy (though not significantly; Table 2). Temperatures and relative humidity (RH) across the classrooms during the school day were quite similar (22.0 ± 1.4 °C and 30.2 ± 10.9%, respectively). At night, temperatures and RH were slightly, but not significantly, lower (20.9 ± 2.5 °C and 29.1 ± 11.5%).

Indoor VOC levels were associated with several classroom characteristics (Table S8). For example, BTEX, terpene and formaldehyde concentrations were positively correlated with the presence of vinyl and wood floor materials, and negatively correlated (along with TTVOCs) with carpeted floors. VOCs other than formaldehyde were associated with the presence of science class materials (e.g., test tubes, animal cages, wood/acrylic cylinders, and science kits) in the classroom. The presence of air fresheners was positively associated with alkane concentrations, and cleaning products with terpene concentrations. Higher BTEX and formaldehyde levels were found in classrooms near bus and vehicle parking areas. These results follow earlier studies, e.g., vinyl flooring and floor adhesives are associated with a variety of aromatic VOCs [11].

No VOC other than benzene and C_11_ showed systematic significant differences by grade level (Table S9). Differences for benzene and C_11_ can be attributed to multiple comparisons. This suggests that emissions and concentrations of the measured VOCs did not vary consistently by grade level, or that changes were too small to observe differences. Studies designed to examine the impact of classroom activities, such as painting, might show impacts (Only one art class was in our sample).

The AERs determined for the classrooms were significantly associated with VOC concentrations. We observed a negative correlation and an inverse relationship (albeit with considerable scatter) for those VOCs with strong indoor sources, e.g., terpenes, and the fitted power law coefficient α in the model C = E·AER^α^/V (C = room air VOC concentration, µg/m^3^; E = emission rate of indoor sources, µg/h; V = room volume, m^3^) was near one, the value expected for pollutants arising from indoor sources (Figure S1). Because HVAC systems were shut down at the end of the school day, AERs dropped considerably in the evening (average of 0.14 ± 0.09 hr^−1^) from school day rates (1.43 ± 1.09 hr^−1^). Correlations were also examined separately for daytime and nighttime AERs. In this case, formaldehyde had the strongest correlation (r = −0.54) with nighttime AERs, possibly reflecting the build-up from slowly emanating sources (e.g., coatings and wood products) when HVAC systems were off. In contrast, terpene levels had the strongest correlation (r = −0.21) with daytime AERs, possibly reflecting the daytime use of wet cleaning products, fragrant candles, and some air fresheners. Plots of VOC concentrations versus AERs (Figure S2) showed that the highest concentrations always occurred in classrooms with the lowest AERs (<1.0 hr^−1^), none of the higher VOC concentrations occurred at high AERs (>3.0 hr^−1^), and VOC levels tended to be low at high AERs (>3.0 hr^−1^). All of this confirms the key role of AERs in determining indoor VOC levels. The low DF of formaldehyde led to a higher R^2^ (0.20) than the others.

ASHRAE 62.1 recommends a minimum outdoor air rate (accounting for both people- and area-related sources) for classrooms of 7.4 L·s^−1^ per person for children aged five to eight years, and 6.7 L·s^−1^ per person for ages of nine years and over using default occupancy densities specified in the standard [23]. Given the occupancies, room sizes, and grades of the tested classrooms, and assuming ideal air distribution and system ventilation efficiency, these requirements represent an average AER of 2.2 ± 0.6 h^−1^. With a more realistic zone air distribution effectiveness of 0.8 (typically from 0.5 to 1.2 depending on the air distribution configuration) and a system ventilation efficiency of 0.8 (typically from 0.6 to 1.0), the required AER would be much higher, 3.6 h^−1^. As mentioned, AERs during the school day averaged 1.4 ± 1.1 h^−1^, considerably lower than recommended. Low AERs have been shown in other school studies [11,14,31]. Here we show that low AERs are associated with higher VOC concentrations, which is consistent with both the user-associated and the emanating types of VOC sources that are present in schools.

The teacher comfort and perception survey had several questions addressing VOCs (Table S8). Although the sample size of this analysis was limited, and only 89 teachers both completed the survey and had IEQ measurements taken in their classroom, statistically significant associations were seen for several responses, e.g., “cleaner” classrooms were associated with lower formaldehyde levels (β = −0.17 ± 0.07, *p* = 0.02), and frequently experienced asphalt odors were associated with higher concentrations of alkanes (β = 0.85 ± 0.30, *p* = 0.01) and TTVOCs (β = 1.17 ± 0.38, *p* = 0.00).

### 4.2. Within- and Between-School Comparisons

The variance analysis indicated that between-school variations in VOC levels exceeded within-school variations for eight compounds (ethylbenzene, xylene, α-pinene, *d*-limonene, 1,2,4-TMB, DCM, and formaldehyde; Table 3). For TTVOCs, 64% of the variation was due to between-school differences. In contrast, benzene, toluene, most alkanes, and other VOCs exhibited greater variability within schools. This variation is important, and may not be adequately recognized and understood. Between-school variation results from factors that either increase or decrease VOC levels across all or most classrooms in the school, e.g., differences due to a school’s location, design, HVAC system type and operation, common building materials, and common building maintenance practices and products. In contrast, within-school (room-to-room) variation mostly results from differences between internal emission sources (also associated with occupant behaviors) and VRs (Ventilation rates) among the classrooms. Within-school variation may be enhanced in classrooms that utilize unit ventilators (UVs), which tend to limit air mixing between different rooms. Of the study schools, nine (24%) employed either UVs or geothermal pumps with UVs, and two (6%) used a mix of AHU and UVs. The remaining 26 schools (70%) had central AHUs that would mix air from multiple rooms, tending to reduce (but not eliminate) room-to-room differences. However, the effectiveness of central AHUs in this regard is diminished with the low ventilation AERs found. When within-school variance is large, it is more difficult to characterize IEQ, and samples must be obtained from multiple rooms within the building [14].

### 4.3. Seasonal Variation

**Figure 3:**
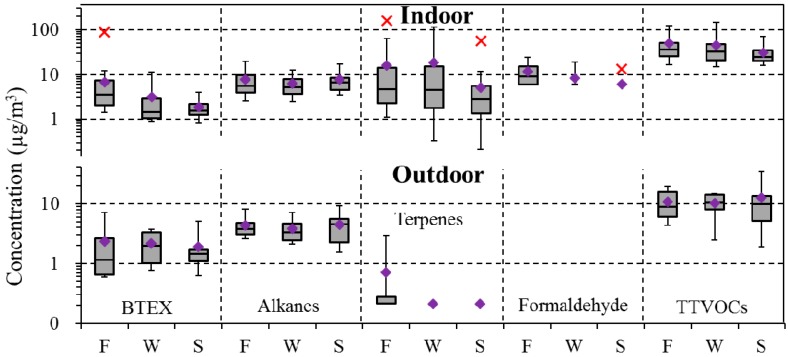
Box plots of BTEX, alkane, terpene, formaldehyde, and TTVOC concentrations indoors and outdoors in three seasons: fall (F), winter (W), and spring (S). Plots show 5th, 25th, 50th, 75th and 95th percentile concentrations. Diamond (♦) denotes mean. Asterisk (x) denotes outliers.

Outdoor concentrations of several VOCs varied by season, e.g., BTEX tended to increase in fall and winter, and decrease in spring (significantly for *p,m*-xylene), while terpenes increased in fall (significantly for α-pinene; Figure 3; Table S10). The study region has a temperate climate with large seasonal changes in temperature that determines heating and cooling needs. (Outdoor temperatures at the study schools averaged 16.7 ± 3.4 °C in fall, 6.3 ± 5.9 °C in winter, and 12.4 ± 4.4 °C in spring; the relative humidity was 52.8 ± 11.7% in fall, 56.3 ± 10.1% in winter, 56.5 ± 14.5% in spring. Indoor temperatures and relative humidities were not correlated with outdoor levels.) Concentrations of aromatic VOCs have been seen to increase in cold weather [32,33,34,35] for several reasons, e.g., lower temperatures reduce photochemical reaction rates and increase atmospheric lifetime [34,36], and biofuel and biomass burning is more widespread in winter [35]. In contrast, emissions of monoterpenes emitted by vegetation are light- and temperature-dependent, and typically are highest in heat, drought and light [33,34,37,38].

Trends indoors depended on the VOC (Figure 3). BTEX concentrations were highest in fall, terpene levels were highest in fall and winter, and alkane levels had only modest changes. Levels of formaldehyde and TTVOC decreased from fall to winter to spring. These differences were statistically significant for benzene, toluene, ethylbenzene, xylene, 1,2,4-TMB, BTEX, C_7_, C_12_, C_14_–C_16_, MCH, *d*-limonene, terpenes, MIBK, *p*-DCB, and formaldehyde (ANOVA and Kruskal-Wallis tests). Most of these patterns followed Jia, Batterman and Godwin [32], and can be attributed to higher indoor temperatures (~0.5 °C higher) which increase internal emissions. However, our ability to examine seasonal factors is limited given that the study did not utilize repeated (or seasonal) measurements at each site. For this reason, the seasonal variation apparent may, in part, reflect site locations or community factors.

### 4.4. Community Variation

**Figure 4:**
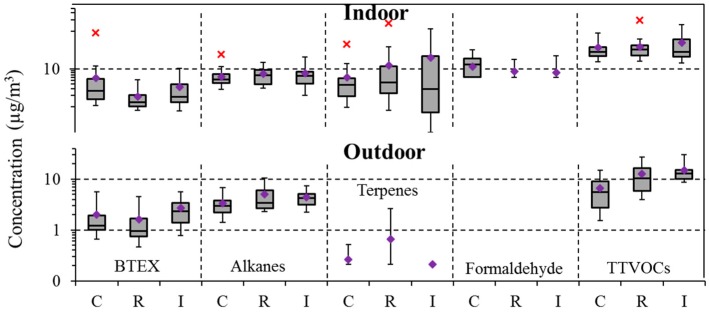
Box plots of BTEX, alkane, terpene, formaldehyde, and TTVOC concentrations indoors and outdoors by community type: no-local emission source or clear (C), road (R), and industrial (I). Plots show 5th, 25th, 50th, 75th and 95th percentile concentrations. Diamond (♦) denotes mean. Asterisk (x) denotes outliers.

We grouped each school’s outdoor environment into one of three broad categories: (1) “no major emission sources” (non-road and non-industrial), (2) “near-road” (highway/freeway/major road/fly-path/railway) community, and (3) “industrial” (near factory/warehouse/brownfield/plowed field/livestock). Simpler urban/rural distinctions were not useful, e.g., the no-emission environments included urban, suburban, town, and city spaces, while the industrial environments included both rural and suburban communities. Based on median concentrations, outdoor alkane and TTVOC levels were highest at the industrial sites, followed by the near-road community and clear environments (Figure 4), with patterns shown elsewhere at industrial and commercial sites [34,39,40]. Perhaps surprisingly, the lowest BTEX levels were found at the near-road schools, however, VOC emissions at highway speeds are now well controlled, e.g., Kimbrough et al. [41] measured concentrations of BTEX and other VOCs near highways and major roads and found larger contributions from parking lots than the highway. VOC emissions may increase when starting and idling vehicles, especially when cold. Additional possible explanations include atmospheric reactions of emitted BTEX, e.g., toluene was the largest contributor to ozone production through atmospheric reactions [42], and the influence of temperature and meteorology on passive sampling rates [29].

Indoor concentrations of BTEX, alkanes and TTVOC followed similar trends as the outdoor levels (though levels were higher), indicating the importance of outdoor sources for the BTEX VOCs. Indoor concentrations of BTEX (toluene, ethylbenzene, and *p,m,o*-xylene), alkanes (C_6_–C_7_, C_13_–C_15_), terpenes, and TTVOCs differed among the no-major emission, near-road and industrial categories (Table S11). For the classrooms (27%) near bus idling areas and parking lots, the median indoor BTEX level (2.9 µg/m^3^) was higher than in other classrooms (1.5 µg/m^3^).

### 4.5. Comparison of Conventional and High Performance School Buildings

**Figure 5:**
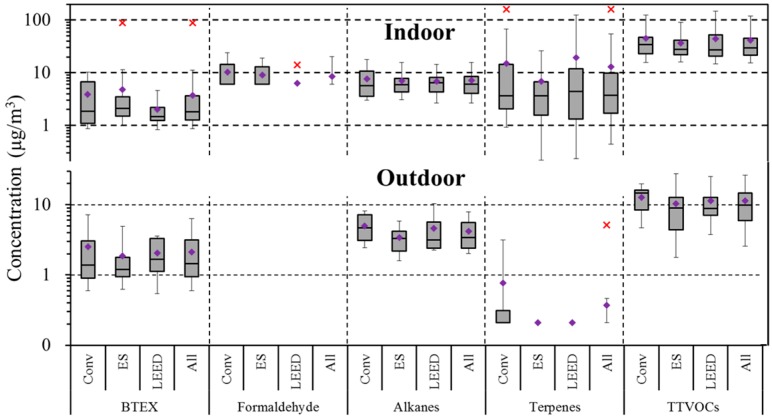
Box plots of indoor and outdoor concentrations of BTEX, formaldehyde, alkanes, terpenes and TTVOCs grouped by three building types: conventional (Conv), EnergyStar (ES), and LEED. Plots show 5th, 25th, 50th, 75th and 95th percentile concentrations. Diamond (♦) shows mean; Crosses (x) show outliers.

Among the three school types, outdoor concentrations of terpenes were higher (*p* = 0.01) at conventional schools, but levels of BTEX, alkanes and TTVOCs did not differ. Indoors, significant differences were seen for benzene, ethylbenzene, xylene, 1,2,4-TMB, BTEX, α-pinene, ethyl acetate, naphthalene, chloroform, formaldehyde and the alkanes group (C_12_–C_16_), although results of ANOVA and K-W tests were not always consistent (Concentrations at conventional, ES and LEED schools, and the t and K-W test results are shown in Table S12). Figure 5 shows box plots by compound group. Overall, concentration differences were not large and no major effect of building type was discerned. This lack of consistent differences might be explained by the similar age and tightness of the buildings, and because the building type itself was associated with other factors that might influence VOC levels. For example, the conventional schools were larger (average area of 95,000 ft^2^) than LEED (73,000 ft^2^) and ES (80,300 ft^2^) schools, all were two-story buildings, and most were situated in urban areas with smaller lots. In contrast, most of the ES and LEED schools were single-story buildings, and many were in agricultural areas or near highways. Moreover, schools within a building type varied in terms of typology, HVAC system design and operation, furnishings, degree of crowding, and other factors. The diversity of school buildings within a building type likely trumps effects due to its classification as a conventional, ES or LEED building. Further stratification of the sample would produce groups too small for robust comparisons.

Another reason why building types may not have differed is that many newer conventional buildings now may be designed very similarly to LEED Silver buildings. Thus, it is possible that differences between building types may only be observed at higher ratings, e.g., LEED Platinum. Our sample numbers, however, were not sufficient to distinguish among levels in the ratings systems.

A key result is that new and recently renovated school buildings and their building systems are diverse and not easily generalized. Unfortunately, data supporting the energy-saving strategies are incomplete, and additional research is needed to link IEQ, energy, ventilation, and the health and performance of students and staff in “green” buildings. Ultimately, such information will make green building certification systems more credible and successful in fostering good IEQ, and will promote the design, construction and operation of schools that are more sustainable.

### 4.6. Comparison with the Literature

Our literature comparisons focus on the more commonly measured VOCs. Table S13 lists indoor and outdoor BTEX and terpene levels measured in the present and earlier school studies. Comparisons must account for regional and building differences, e.g., all schools in the present study were mechanically ventilated, while European studies have mostly examined naturally ventilated schools that usually have lower VRs.

As noted earlier and by Su et al. [43], changes in the ambient concentrations of VOCs will affect indoor levels. Outdoors, concentrations of many VOCs in the U.S. have declined by an estimated 38% from 1990 to 2014 [44], largely due to reductions in emissions of toxics and ozone precursors from transportation, industry and consumer sectors [45,46]. Control measures include, for example, the now-universal use of catalytic converters on gasoline vehicles [47], limits on fuel vapor pressure and benzene content, and the lowered VOC content of paints and coatings [48]. Several studies have confirmed the link between these measures and ambient concentrations, e.g., benzene concentrations declined 88% from 1990 to 2012 in California due to lower industrial emissions [49], and concentrations for a range of VOCs fell from 43% to 72% from 1986 to 2015 in the southern central U.S. due to O_3_ precursor controls [50].

Our indoor results are generally comparable to levels measured in schools in Minneapolis, Minnesota [51], Bari, Italy [13], and Ann Arbor, Michigan [14]. For example, median levels of *d*-limonene and α-pinene (4.4 and 0.1 µg/m^3^, respectively, in winter, and 2.6 and 0.1 µg/m^3^ in spring) measured in this study are similar to levels in Minneapolis schools (4.6 and 0.2 µg/m^3^ in winter, and 1.9 and 0.2 µg/m^3^ in spring). Examining the broader set of studies, VOC levels in U.S. schools have been gradually decreasing over the past two decades, e.g., BTEX levels that averaged 7.2 µg/m^3^ in 2000 [51] fell to 5.7 µg/m^3^ in 2003 [14] and then to only 1.4 µg/m^3^ in the present study. We note that the school literature is relatively small, anomalies exist (e.g., a benzene level of only 0.1 µg/m^3^ was reported in the 2007 Michigan study), some compounds may follow different trends (e.g., concentrations of brominated flame retardants increased greatly from about 1980 to 2010 [52], and trends are best determined using repeated measures. For common VOCs, however, trends in schools appear to parallel the decreasing concentrations found in North American residences from 1990 to 2005 [53], as well as the decreasing VOC exposure in U.S. adults seen from 1988 to 2004 based on blood biomarkers [54]. Altogether, these trends suggest that the newer “environmentally friendly” products and building materials, coupled with reductions in ambient concentrations, have lowered VOC exposure in schools.

### 4.7. Limitations

This study characterized VOC concentrations in 144 school rooms at 37 recently constructed or renovated schools in four Midwest states. Our results may not apply to buildings that are older, naturally ventilated, located in different climatic regimes, or situated in other countries or settings that do not have effective VOC controls or restrictions on indoor and outdoor pollution sources. Many of the measurements took place in cooler weather, and the low AER rates found (likely to save energy costs) may reflect a “worst case” situation. Sampling emphasized regular classrooms, though a few science and art rooms were included. Gyms, science rooms, computer labs, natatoriums, shops, libraries, and other specialized spaces in schools were not included. The walk-through inspections noted potential VOC sources, but did not detail every decoration, personal care products, cleaning products, and other possible VOC sources, and only a qualitative assessment of sources was provided. A larger number of schools and repeated visits would improve the ability to evaluate differences due to school type, though the collected data appear sufficient to characterize VOC levels in schools to evaluate at least some differences, e.g., effect of ventilation.

A few samples (three indoor and two outdoor) were missing due to instrument failure, but this represents a small fraction of the collected data. We measured a wide range of VOCs, but very volatile or semi-volatile compounds, aldehydes other than formaldehyde, and some other compounds of interest were not measured. The detection limits may have been too high to detect some VOCs. Since passive sampling was employed, measurements included two (occupied) school days plus the in-between evening and nighttime period. Generally, the unoccupied period is of less interest for exposure purposes. The one-way tests and correlations examining potential effects of season, building type, location, etc., do not account for possible covariates and confounders, and repeated visits to the same schools would help confirm seasonal variability. The IEQ encompasses additional air pollutants, as well as lighting and noise. These topics will be the focus of subsequent papers. Finally, we note that the sampling method, duration, instruments, and analysis methods for VOC characterizations differ among studies. The one- to two-day sampling period in this study was relatively short, which challenged the method sensitivity, yet quality assurance goals were met and results were comparable to the sampling method (using 31 h passive sampling) in a previous study in the U.S. [51].

## 5. Conclusions

Schools are vital environments due to the amount of time children spend in classrooms and other school settings. Impacts of the school environment on the health and academic performance of students, which are just beginning to be recognized, motivate the need to better characterize environmental conditions in buildings and to understand whether current green building guidelines promote a healthier environment in schools. To date, however, characterizations of IAQ in schools have been limited, and studies comparing VOCs in high performance and conventional buildings are not available.

Indoor and outdoor VOC sampling in 37 recently constructed or renovated schools across the U.S. Midwest was conducted to investigate exposure in schools and potential associations with environmental parameters and building type. Of the 94 target VOCs, we characterized 10 species outdoors and 24 species indoors. Outdoors, benzene, toluene, *p,m*-xylene, C_6_ and chloroform were most abundant with median concentrations from 0.1 to 2.4 µg/m^3^. Indoors, benzene, toluene, *p,m*-xylene, *d*-limonene, and *n*-hexane were most common with median concentrations from 0.3 to 3.5 µg/m^3^. Building inspections suggested several VOC sources, e.g., paints, cleaning products, flooring materials, air fresheners, and industry, and VOC levels were inversely associated with air exchange rates. For many compounds, the within-school variance of VOC concentrations exceeded the between-school variance, indicating the role of local sources, independent ventilation systems, and human activities. The conventional and high performance (EnergyStar and LEED) school buildings did not show systematic differences in VOC levels, likely due to the tremendous diversity of school buildings and their building systems.

Overall, VOC concentrations were mostly low and measured concentrations in this study (2015–2016) appear to have declined from levels measured in previous decades. This suggests the effectiveness of VOC controls on outdoor sources and the widespread use of low-emission materials and products, and that these factors have offset possible increases in concentrations due to the low air exchange rates in new and “tight” buildings. Still, opportunities remain to improve the IAQ by limiting emissions from building-related sources and products (e.g., building materials, cleaning products, pesticides, fragrances), and by increasing ventilation rates.

## Figures and Tables

**Table 1:** Summary statistics of VOC concentrations in schools and outdoors.

VOC	Outdoors (*n* = 35)				Indoors (*n* = 144)				*p*-Value *	
	DF (%)	Mean (µg/m^3^)	Median (µg/m^3^)	Max (µg/m^3^)	DF (%)	Mean (µg/m^3^)	Median (µg/m^3^)	Max (µg/m^3^)	Paired *t*-Test	Signed Rank
Aromatics										
Benzene	86	0.5	0.5	2.1	93	0.5	0.3	4.4	0.90	0.58
Toluene	97	1.0	0.4	7.0	100	1.8	0.7	83.8	0.22	**0.01**
Ethylbenzene	17	0.1	0.1	0.9	36	0.2	0.1	2.0	**0.05**	**0.00**
*p,m*-Xylene	46	0.4	0.1	3.6	81	0.9	0.4	7.4	**0.01**	**0.00**
*o*-Xylene	23	0.1	0.1	1.3	51	0.3	0.1	2.2	**0.01**	**0.00**
1,2,4-Trimethylbenzene	29	0.2	<0.03	3.8	59	0.3	0.2	4.2	0.22	**0.00**
BTEX	97	2.0	1.3	8.5	100	3.7	1.8	89.1	**0.05**	**0.01**
Alkanes										
*n*-Hexane	97	3.4	2.4	12.9	95	3.2	2.2	27.2	0.86	0.65
*n*-Heptane	9	0.2	0.1	2.3	35	0.9	0.1	9.9	**0.00**	**0.00**
*n*-Undecane	0	<0.06	<0.06	<0.06	17	0.3	0.1	4.3	**0.01**	**0.00**
*n*-Dodecane	11	0.2	0.2	0.5	51	0.6	0.3	3.0	**0.00**	**0.00**
*n*-Tridecane	3	0.1	0.1	0.5	17	0.2	0.1	1.5	**0.01**	**0.01**
*n*-Tetradecane	6	0.1	0.1	0.3	85	1.3	1.0	8.2	**0.00**	**0.00**
*n*-Pentadecane	0	<0.04	<0.04	<0.04	35	0.5	<0.04	5.8	**0.00**	**0.00**
*n*-Hexadecane	0	<0.06	<0.06	<0.06	28	0.3	0.1	2.6	**0.00**	**0.00**
Methyl cyclohexane	15	2.1	1.3	11.8	28	3.0	1.3	53.1	0.20	0.13
Alkanes (C_6_–C_7_, C_11_–C_16_)	97	4.0	3.4	13.5	99	7.2	6.1	28.8	**0.00**	**0.00**
Terpenes										
α-Pinene	6	0.2	0.1	5.0	47	1.5	0.1	55.7	0.19	**0.00**
*d*-Limonene	3	0.1	0.1	0.7	94	11.3	3.5	158.5	**0.00**	**0.00**
Terpenes	9	0.3	0.2	5.1	95	12.8	3.8	159.7	**0.00**	**0.00**
Other										
Ethyl acetate	0	<0.25	<0.25	<0.25	26	0.8	0.3	7.9	**0.00**	**0.00**
Methyl isobutyl ketone	0	<0.04	<0.04	<0.04	24	0.2	<0.04	4.3	**0.01**	**0.00**
Naphthalene	11	0.2	0.1	1.7	40	0.2	0.1	2.9	0.28	**0.00**
Methylene chloride	29	2.4	0.8	13.9	20	2.8	0.8	47.7	0.65	1.00
Chloroform	37	2.3	0.1	19.8	31	1.7	0.1	15.3	0.42	0.71
1,4-Dichlorobenzene	0	<0.03	<0.03	<0.03	15	0.1	<0.03	2.9	**0.04**	**0.00**
Formaldehyde	-	-	-	-	23	8.6	6.0	32.0	-	-
TTVOCs	100	24.2	21.6	76.5	100	41.5	29.3	196.6	**0.00**	**0.00**

* *p*-value test for the difference between indoor and outdoor VOCs. Bold values are statistically significant (*p* < 0.05). DF: detection frequency.

**Table 2:** Spearman rank correlation coefficients of indoor VOC concentrations and key IEQ parameters (*n* = 144).

VOC	Benzene	Toluene	Ethyl-Benzene	*m,p*-Xylene	*o*-Xylene	BTEX	Alkanes	Terpenes	Formaldehyde	TTVOCs	Occupancy	Rh (%)	Avg. CO_2_	Daytime Avg. CO_2_	Avg. Aer	Daytime Avg. Aer	Nighttime Avg. Aer
Benzene	1.000																
Toluene	0.133	1.000															
Ethylbenzene	0.460 *	0.427 *	1.000														
*m,p*-Xylene	0.363 *	0.586 *	0.985 *	1.000													
*o*-Xylene	0.450 *	0.452 *	0.940 *	0.938 *	1.000												
BTEX	0.250 *	0.857 *	0.908 *	0.839 *	0.819 *	1.000											
Alkanes	0.143	0.368 *	0.269 *	0.238 *	0.178	0.330 *	1.000										
Terpenes	0.175 *	0.317 *	0.303 *	0.354 *	0.328 *	0.354 *	0.425 *	1.000									
Formaldehyde	0.178 *	0.245 *	0.529 *	0.283 *	0.340 *	0.275 *	0.028	0.353 *	1.000								
TTVOCs	0.222 *	0.466 *	0.472 *	0.484 *	0.493 *	0.555 *	0.617 *	0.753 *	0.561 *	1.000							
Occupancy	−0.086	0.041	−0.145	−0.181	−0.214	−0.037	−0.022	0.058	0.082	0.053	1.000						
RH (%)	−0.011	0.331 *	0.588 *	0.428 *	0.440 *	0.405 *	0.129	0.332 *	0.490 *	0.399 *	−0.089	1.000					
Avg. CO_2_	−0.116	0.370 *	0.185	0.169	0.110	0.296 *	0.452 *	0.397 *	0.260 *	0.416 *	0.326 *	0.423 *	1.000				
Daytime avg. CO_2_	−0.019	0.316 *	0.305 *	0.183	0.183	0.233 *	0.109	0.414 *	0.470 *	0.448 *	0.179 *	0.411 *	0.506 *	1.000			
Avg. AER	−0.035	−0.132	−0.153	−0.149	−0.239 *	−0.167 *	−0.106	−0.202 *	−0.363 *	−0.238 *	0.137	−0.225 *	−0.285 *	−0.154	1.000		
Day avg. AER	−0.034	−0.126	−0.136	−0.128	−0.206 *	−0.174 *	−0.074	−0.208 *	−0.307 *	−0.238 *	0.125	−0.220 *	−0.262 *	−0.164	0.993 *	1.000	
Night avg. AER	0.047	−0.132	−0.130	−0.204 *	−0.213 *	−0.152	−0.125	−0.135	−0.541 *	−0.123	0.153	−0.217 *	−0.150	0.146	0.310 *	0.227 *	1.000

Occupancy is the average number of teachers and students. RH is the relative humidity (%). AER is the air exchange rate, measured over the school day (daytime) and at nighttime. * Bold values are statistically significant (*p* < 0.05).

**Table 3:** Within- and between-school variation in indoor VOC concentrations.

VOC	Percent of Variation (%)		*p*-Value *
	Within-School	Between-School	
Aromatics			
Benzene	89.1	10.9	0.06
Toluene	91.8	8.2	0.11
Ethylbenzene	14.2	85.8	**0.00**
*p,m*-Xylene	16.5	83.5	**0.00**
*o*-Xylene	28.6	71.4	**0.00**
1,2,4-TMB	41.7	58.3	**0.00**
BTEX	83.6	16.4	**0.01**
Alkanes			
C6	82.0	18.0	**0.01**
C7	60.9	39.1	**0.00**
C11	62.4	37.6	**0.00**
C12	70.0	30.0	**0.00**
C13	77.9	22.1	**0.00**
C14	82.2	17.8	**0.00**
C15	59.2	40.8	**0.00**
C16	73.3	26.7	**0.00**
MCH	84.3	15.7	**0.01**
Alkanes	69.8	30.2	**0.00**
Terpenes			
α-Pinene	15.7	84.3	**0.00**
*d*-Limonene	36.1	63.9	**0.00**
Terpenes	25.6	74.4	**0.00**
Other			
Ethyl acetate	58.0	42.0	**0.00**
MIBK	57.5	42.5	**0.00**
Naphthalene	76.7	23.3	**0.00**
DCM	48.6	51.4	**0.00**
Chloroform	52.1	47.9	**0.00**
*p*-DCB	63.2	36.8	**0.00**
Formaldehyde	37.2	62.8	**0.00**
TTVOCs	35.4	64.6	**0.00**

Bold values are statistically significant (*p* < 0.05). * *p*-value test for the VOC differences between-schools.

## Supplementary Materials

The following are available online at www.mdpi.com/1660-4601/14/1/100/s1, Table S1: Studies examining VOCs in schools in the literature, Table S2: School building characterization, Table S3: Target compounds and analytical performance, Table S4: Ninety-four GC/MS calibration compounds and detection frequency (DF), Table S5: Spearman rank correlation coefficient for indoor and outdoor BTEX, Table S6: Spearman rank correlation coefficient for indoor and outdoor alkanes, Table S7: Spearman rank correlation coefficient for indoor and outdoor terpenes, Table S8: Associations between VOCs and classroom characteristics/teacher survey in schools in the present study, Table S9: Statistics of indoor VOC concentrations by grade in schools in the present study, Table S10: Statistics of VOC concentrations measured indoors and outdoors by season in schools in the present study, Table S11: Statistics of VOC concentrations measured indoors and outdoors by dominant identified community outdoor source type in schools in the present study, Table S12: Comparison of indoor and outdoor VOC concentrations by building type in schools in the present study, Table S13: Comparison of indoor and outdoor VOC concentrations in school studies, Figure S1: One-zone IAQ model, Figure S2: Indoor measured concentrations of terpenes, BTEX, formaldehyde, and TTVOCs against average AERs.
