# Supplementary material for: Volatile Organic Compounds (VOCs) in Conventional and High Performance School Buildings in the U.S
Source: Int J Environ Res Public Health. 2017 Jan 21;14(1):100. doi: 10.3390/ijerph14010100 (PMC5295350; doi:10.3390/ijerph14010100)
Supplement: Supplementary file 1 [file ijerph-14-00100-s001.pdf]

# Supplementary Materials: Volatile Organic Compounds (VOCs) in Conventional and High Performance School Buildings in the U.S.

**Lexuan Zhong, Feng-Chiao Su and Stuart Batterman ***

**Table S1.** Studies examining VOCs in schools in the literature.

| Papers | No. of Schools | No. of Classrooms | Location | Sampling Method | Analysis Method | Major Remarks |
|---|---|---|---|---|---|---|
| [1] | 6 | 97 | Sweden | Active sampling (1 L/min, 2 hr), charcoal tubes | Extracted with $CS_2$ and GC/MS | Perception of high room temperature and air dryness were related to high VOC concentration. |
| [2] | 7 | 20 | Los Angeles, USA | Passive sampling (5–7 days), charcoal tubes | Extracted with acetone and $CS_2$, GC/MS | The four most prevalent VOCs measured were toluene, *p*/*m*-xylene, $\alpha$-pinene, and limonene. |
| [3] | 2 | 10 | Minnesota, USA | Passive sampling (indoor: 31 hr; outdoor: 103 hr), charcoal-based tubes | Extracted with acetone and $CS_2$ and GC/MS | The school and outdoor environments had similar influence on personal exposure to VOCs. |
| [4] | 2 | 4 | California, USA | Active sampling (5-6 mL/min for VOCs, 150 mL/min for aldehydes, 7–8 h), modified Tenax-TA tubes, DNPH tubes | TD/GC/MS | Indoor minus outdoor VOC concentrations with an advanced HVAC operated were low; only formaldehyde concentrations exceeded 5 ppb. |
| [5] | 9 | 64 | Michigan, USA | Passive sampling (3.5–4.5 days), Tenax GR tubes | Thermal desorption (TD)/cryofocusing system and GC/MS | Art room, science room, and indoor pools are identified VOC sources. |
| [6] | 3 | - | Kocaeli, Turkey | Passive sampling (1 day), graphitized charcoal tubes (35–50 mesh) | TD/GC/FID | Indoor concentrations were generally higher than those observed outdoors. Indoor target compound concentrations were more strongly correlated with outdoor concentrations in summer than in winter. |
| [7] | 9 | 18 | Porto, Portugal | Low flow sample pump, Tenax-TA tubes | GC/MS | No statistically significant association between asthma symptoms and VOC concentrations. |

**Table S1.** *Cont.*

| Papers | No. of Schools | No. of Classrooms | Location | Sampling Method | Analysis Method | Major Remarks |
|---|---|---|---|---|---|---|
| [8] | - | 46–188 | 11 European cities | Passive sampling (7 days), charcoal tubes, DNPH tubes | Extracted with $CS_2$ and GC/MS, HPLC | Sources in the indoor environment are prevailing for most VOCs. Aromatics in indoors were almost equal to the outdoors. Terpenes were low during warm seasons. |
| [9,10] | 14 | 28 | Lisbon, Portugal | Passive sampling (14 days), activated charcoal (35–50 mesh) tubes, DNPH tubes | Extracted with $CS_2$ and GC/FID; HPLC | I/O ratios above unity indicate the impact of indoor sources and building conditions on IAQ. Higher indoor VOC concentrations occur often in the winter, and carbonyl concentrations were higher in the summer. |
| [11] | 109 | More than 400 | 6 French cities | Passive sampling | - | Formaldehyde, acetaldehyde and acrolein showed low to moderate degree of similarity between the classrooms. |
| [12] | 8 | 24 | Italy | Passive sampling (5 days), Carbograph tubes (35–50 mesh) | TD/GC/MS | High terpenes were found in all monitored classroom. Outdoor concentrations were lower than indoors for each monitored school. |
| [13] | 2 | 6 | London, UK | Passive sampling (5 days) | TD/GC/MS | VOC concentrations in the classrooms were much higher than outdoor levels. |
| [14] | 20 | 73 | Portugal | Passive sampling (5 days), Tenax TA (60/80 mesh) tubes | TD/GC/MS | The median TVOC was 140.3 μg/m$^3$, which was lower than the WHO (2010) values. |

VOC: volatile organic compounds; DNPH: 2,4-Dinitrophenylhydrazine; TA: trapping Agent; GR: graphite; GC/MS: gas chromatograph/mass spectrometer; FID: flame ionization detector; HPLC: high performance liquid chromatography; HVAC: heating, ventilation and air conditioning; I/O: indoor/outdoor; IAQ: indoor air quality; TVOC: total target VOC.

**Table S2.** School building characterization.

| Energy Performance | Conventional | Energy Star | LEED | |
|---|---|---|---|---|
| Count | 10 | 15 | 12 | |
| Building typology | Single-story | Two-story | Three-story | |
| Count | 17 | 14 | 6 | |
| Ventilation system | AHU | UV | AHU + UV | Geothermal pump |
| Count | 26 | 4 | 2 | 5 |
| Filter | MERV-8 | MERV-higher level | other | |
| Count | 15 | 17 | 5 | |
| Classroom | General classroom | Music room | Art room | Resource room |
| Count | 139 | 2 | 1 | 2 |

LEED: leadership in energy and environmental design; AHU: air handling units; UV: unit ventilator; MERV: minimum efficiency reporting value.

**Table S3.** Target compounds and analytical performance.

| Compound | Abbreviation | CAS No. | Target Ion | Qualifier Ion | MDL (ug/m³) |
|---|---|---|---|---|---|
| Aromatics | | | | | |
| Benzene | | 71-43-2 | 78 | 50 | 0.08 |
| Toluene | | 108-88-3 | 91 | 92 | 0.07 |
| Ethylbenzene | | 100-41-4 | 91 | 106 | 0.14 |
| *p,m*-Xylene | | 106-42-3, 108-38-3 | 91 | 106 | 0.16 |
| *o*-Xylene | | 95-47-6 | 91 | 106 | 0.09 |
| 1,2,4-trimethylbenzene | 1,2,4-TMB | 95-63-6 | 105 | 120 | 0.06 |
| Alkanes | | | | | |
| *n*-Hexane | $C_6$ | 110-54-3 | 57 | 86 | 0.19 |
| *n*-Heptane | $C_7$ | 142-82-5 | 43 | 71 | 0.24 |
| *n*-Undecane | $C_{11}$ | 1120-21-4 | 57 | 43 | 0.11 |
| *n*-Dodecane | $C_{12}$ | 112-40-3 | 57 | 43 | 0.44 |
| *n*-Tridecane | $C_{13}$ | 629-50-5 | 57 | 43 | 0.15 |
| *n*-Tetradecane | $C_{14}$ | 629-62-9 | 57 | 43 | 0.13 |
| *n*-Pentadecane | $C_{15}$ | 544-76-3 | 57 | 43 | 0.08 |
| *n*-Hexadecane | $C_{16}$ | 536-78-7 | 105 | 104, 78 | 0.13 |
| Methyl cyclohexane | MCH | 108-87-2 | 83 | 55 | 2.50 |
| Terpenes | | | | | |
| $\alpha$-Pinene | | 7785-70-8 | 93 | 77, 105 | 0.17 |
| *d*-Limonene | | 5989-27-5 | 68 | 93, 136 | 0.25 |
| Other | | | | | |
| Ethyl acetate | | 141-78-6 | 43 | 45 | 0.50 |
| Methyl isobutyl ketone | MIBK | 108-10-1 | 43 | 58, 55, 100 | 0.08 |
| Naphthalene | | 91-20-3 | 128 | 102 | 0.24 |
| Methyl chloride | DCM | 75-09-2 | 84 | 49 | 0.75 |
| Chloroform | | 67-66-3 | 83 | 47 | 0.25 |
| 1,4-Dichlorobenzene | *p*-DCB | 541-73-1 | 146 | 111, 75 | 0.06 |
| Formaldehyde | | 50-00-0 | N/A | N/A | 6.00 |

MDL: Method detection limits.

**Table S4.** Ninety-four GC/MS calibration compounds and detection frequency (DF).

| No. | VOC | DF (%) | No. | VOC | DF (%) |
|---|---|---|---|---|---|
| 1 | 1,2-Dichloroethylene (trans, E) | 3 | 48 | Tetrachloroethene | 8 |
| 2 | Methylene chloride | 20 | 49 | 1,1,1,2-Tetrachloroethane | 0 |
| 3 | Hexane | 95 | 50 | Chlorobenzene | 0 |
| 4 | Methyl t-butyl ether | 0 | 51 | Ethylbenzene | 36 |
| 5 | 1,1-Dichloroethane | 0 | 52 | *p*-Xylene, *m*-Xylene | 81 |
| 6 | Propanenitrile | 0 | 53 | Bromoform | 0 |
| 7 | Butanal | 0 | 54 | *n*-Nonane | 6 |
| 8 | 2-Butanone | 3 | 55 | Heptanal | 0 |
| 9 | Methacrylonitrile | 0 | 56 | Styrene | 10 |
| 10 | 1,2-Dichloroethylene (Cis, Z) | 0 | 57 | 1,1,2,2-Tetrachloroethane | 1 |
| 11 | Bromochloromethane | 0 | 58 | *o*-Xylene | 51 |
| 12 | Chloroform | 31 | 59 | 1,2,3-Trichloropropane | 0 |
| 13 | Ethyl acetate | 26 | 60 | 1,4-Dichlor-2-butene (trans, E) | 0 |
| 14 | 2,2-Dichloropropane | 0 | 61 | Isopropylbenzene (cumene) | 0 |
| 15 | Methyl acrylate | 0 | 62 | a-Pinene (1R) - (+) | 47 |
| 16 | Tetrahydrofuran | 1 | 63 | Bromobenzene | 0 |
| 17 | 1,2-Dichloroethane | 1 | 64 | *n*-Propylbenzene | 0 |
| 18 | 1,1,1-Trichloroethane | 0 | 65 | 2-Chlorotoluene | 0 |
| 19 | Butyl chloride | 0 | 66 | 4-Chlorotoluene | 0 |
| 20 | 1,1-Dichloropropene | 0 | 67 | 4-Ethyl toluene | 2 |
| 21 | Chloroacetonitrile | 0 | 68 | 1,3,5-Trimethylbenzene | 10 |
| 22 | Isopropyl acetate | 0 | 69 | Pentachloroethane | 0 |
| 23 | Carbontetrachloride | 0 | 70 | *n*-Decane | 8 |
| 24 | Benzene | 93 | 71 | tert-Butylbenzene | 0 |
| 25 | pentanal | 0 | 72 | 1,2,4-Trimethylbenzene | 59 |
| 26 | 2-nitropropane | 0 | 73 | sec-Butylbenzene | 0 |
| 27 | Dibromomethane | 0 | 74 | 1,3-Dichlorobenzene | 0 |
| 28 | n-Heptane | 35 | 75 | 1,4-Dichlorobenzene | 15 |
| 29 | 1,2-Dichloropropane | 0 | 76 | p-Isopropyltoluene | 1 |
| 30 | Trichloroethylene | 1 | 77 | Limonene (R) - (+) | 94 |
| 31 | Bromodichloromethane | 0 | 78 | o-Cresol | 0 |
| 32 | Methyl methacrylate | 0 | 79 | 1,2-Dichlorobenzene | 0 |
| 33 | *n*-propyl acetate | 0 | 80 | *p,m*-Cresol | 0 |
| 34 | Methyl cyclohexane | 28 | 81 | *n*-Butylbenzene | 0 |
| 35 | 1,1-Dichloro-2-propanone | 0 | 82 | n-Undecane | 17 |
| 36 | 1,3-Dichloropropene (Cis, Z) | 0 | 83 | 1,2-Dibromo-3-chloropropane | 0 |
| 37 | 1,3-Dichloropropene (trans, E) | 0 | 84 | Hexachloroethane | 0 |
| 38 | 1,1,2-Trichloroethane | 0 | 85 | Nitrobenzene | 0 |
| 39 | Toluene | 100 | 86 | *n*-Dodecane | 51 |
| 40 | 1,3-Dichloropropane | 0 | 87 | 1,2,4-Trichlorobenzene | 0 |
| 41 | Ethyl methacrylate | 0 | 88 | Naphthalene | 40 |
| 42 | Methyl isobutyl ketone | 24 | 89 | Hexachlorobutadiene | 0 |
| 43 | 2-Hexanone | 0 | 90 | 1,2,3-Trichlorobenzene | 0 |
| 44 | Dibromochloromethane | 0 | 91 | *n*-Tridecane | 17 |
| 45 | *n*-Octane | 12 | 92 | *n*-Tetradecane | 85 |
| 46 | Hexanal | 0 | 93 | *n*-Pentadecane | 35 |
| 47 | 1,2-Dibromoethane | 0 | 94 | *n*-Hexadecane | 28 |

**Table S5.** Spearman rank correlation coefficient for indoor and outdoor BTEX.

| VOC | Benzene | Toluene | Ethylbenzene | *p,m*-Xylene | *o*-Xylene | BTEX | TTVOCs |
|---|---|---|---|---|---|---|---|
| Benzene | 1.000 | | | | | | |
| Toluene | 0.133 | 1.000 | | | | | |
| Ethylbenzene | 0.460 ** | 0.427 ** | 1.000 | | | | |
| *p,m*-Xylene | 0.363 ** | 0.586 ** | 0.985 ** | 1.000 | | | |
| *o*-Xylene | 0.450 ** | 0.452 ** | 0.940 ** | 0.938 ** | 1.000 | | |
| BTEX | 0.250 ** | 0.857 ** | 0.908 ** | 0.839 ** | 0.819 ** | 1.000 | |
| TTVOCs | 0.222 ** | 0.466 ** | 0.472 ** | 0.484 ** | 0.493 ** | 0.555 ** | 1.000 |

** Denotes $p < 0.01$; BTEX: Benzene, toluene, ethylbenzene and xylene.

**Table S6.** Spearman rank correlation coefficient for indoor and outdoor alkanes.

| | $C_6$ | $C_7$ | $C_{11}$ | $C_{12}$ | $C_{13}$ | $C_{14}$ | $C_{15}$ | $C_{16}$ | Alkanes | TTVOCs |
|---|---|---|---|---|---|---|---|---|---|---|
| $C_6$ | 1.000 | | | | | | | | | |
| $C_7$ | −0.030 | 1.000 | | | | | | | | |
| $C_{11}$ | −0.032 | 0.293 ** | 1.000 | | | | | | | |
| $C_{12}$ | 0.009 | −0.042 | 0.157 | 1.000 | | | | | | |
| $C_{13}$ | −0.031 | −0.048 | 0.063 | 0.390 ** | 1.000 | | | | | |
| $C_{14}$ | 0.035 | −0.122 | 0.086 | 0.334 ** | 0.109 | 1.000 | | | | |
| $C_{15}$ | 0.105 | −0.020 | −0.070 | 0.244 ** | 0.288 ** | 0.365 ** | 1.000 | | | |
| $C_{16}$ | −0.028 | −0.088 | 0.066 | 0.450 ** | 0.237 ** | 0.333 ** | 0.498 ** | 1.000 | | |
| Alkanes | 0.517 ** | 0.287 ** | 0.278 ** | 0.378 ** | 0.280 ** | 0.457 ** | 0.440 ** | 0.379 ** | 1.000 | |
| TTVOCs | 0.312 ** | 0.224 ** | 0.375 * | 0.374 ** | 0.297 ** | 0.321 ** | 0.327 ** | 0.272 ** | 0.617 ** | 1.000 |

** Denotes $p < 0.01$; * Denotes $p < 0.05$.

**Table S7.** Spearman rank correlation coefficient for indoor and outdoor terpenes.

| VOC | $\alpha$-Pinene | *d*-Limonene | Terpenes | TTVOCs |
|---|---|---|---|---|
| $\alpha$-Pinene | 1.000 | | | |
| *d*-Limonene | 0.512 ** | 1.000 | | |
| Terpenes | 0.595 ** | 0.986 ** | 1.000 | |
| TTVOCs | 0.439 ** | 0.751 ** | 0.745 ** | 1.000 |

** Denotes $p < 0.01$.

**Table S8.** Associations between VOCs and classroom characteristics/teacher survey in schools in the present study.

| Variable | Rooms/Group | BTEX | | | Alkanes | | | Terpenes | | | Formaldehyde | | | TTVOCs | | |
|---|---|---|---|---|---|---|---|---|---|---|---|---|---|---|---|---|
| | | β [1] | SE [2] | *p* [3] | β [1] | SE [2] | *p* [3] | β [1] | SE [2] | *p* [3] | β [1] | SE [2] | *p* [3] | β [1] | SE [2] | *p* [3] |
| Floor materials | | | | | | | | | | | | | | | | |
| Vinyl | 75 | 0.45 | 0.14 | **0.00** | 0.07 | 0.10 | 0.47 | 0.56 | 0.26 | **0.03** | 0.37 | 0.07 | **0.00** | 0.12 | 0.13 | 0.35 |
| Carpet | 85 | −0.59 | 0.14 | **0.00** | −0.16 | 0.10 | 0.12 | −1.59 | 0.23 | **0.00** | −0.34 | 0.08 | **0.00** | −0.59 | 0.12 | **0.00** |
| Rugs | 36 | 0.39 | 0.16 | **0.02** | 0.05 | 0.11 | 0.68 | 0.45 | 0.29 | 0.12 | 0.16 | 0.09 | 0.07 | 0.10 | 0.14 | 0.49 |
| Wood | 37 | 0.52 | 0.16 | **0.00** | 0.20 | 0.11 | 0.07 | 0.66 | 0.28 | **0.02** | 0.42 | 0.08 | **0.00** | 0.23 | 0.14 | 0.11 |
| Plastic | 46 | 0.48 | 0.15 | **0.00** | 0.05 | 0.10 | 0.65 | 0.50 | 0.27 | 0.06 | 0.23 | 0.08 | **0.01** | 0.09 | 0.13 | 0.49 |
| Metal | 44 | 0.71 | 0.14 | **0.00** | 0.17 | 0.10 | 0.11 | 0.98 | 0.26 | **0.00** | 0.46 | 0.07 | **0.00** | 0.36 | 0.13 | **0.01** |
| Chemical sources | | | | | | | | | | | | | | | | |
| Science supply | 2 | 1.33 | 0.58 | **0.03** | 0.60 | 0.40 | 0.14 | 2.36 | 1.05 | **0.03** | 0.52 | 0.32 | 0.12 | 1.31 | 0.51 | **0.01** |
| Cleaning supply | 51 | −0.02 | 0.15 | 0.92 | 0.05 | 0.10 | 0.65 | 0.45 | 0.26 | 0.09 | 0.04 | 0.08 | 0.66 | 0.19 | 0.13 | 0.16 |
| Air freshener | 34 | −0.07 | 0.17 | 0.66 | 0.27 | 0.11 | **0.02** | 0.18 | 0.30 | 0.55 | −0.02 | 0.09 | 0.80 | 0.12 | 0.15 | 0.43 |
| Plants/animals | | | | | | | | | | | | | | | | |
| Plants | 14 | −0.11 | 0.24 | 0.64 | −0.20 | 0.16 | 0.21 | 0.47 | 0.42 | 0.26 | 0.33 | 0.13 | **0.01** | −0.02 | 0.21 | 0.92 |
| Pet-open habitat | 10 | −0.29 | 0.27 | 0.29 | 0.12 | 0.19 | 0.54 | 1.24 | 0.48 | **0.01** | 0.21 | 0.15 | 0.16 | 0.42 | 0.24 | 0.08 |
| Pet-close habitat | 4 | 0.31 | 0.42 | 0.47 | 0.25 | 0.29 | 0.39 | 1.12 | 0.75 | 0.14 | −0.25 | 0.23 | 0.28 | 0.93 | 0.37 | **0.01** |
| Near emissions | | | | | | | | | | | | | | | | |
| Mechanical (inside) | 1 | 0.19 | 0.84 | 0.83 | 0.22 | 0.57 | 0.70 | 3.4 | 1.5 | 0.02 | −0.25 | 0.46 | 0.60 | 1.78 | 0.73 | **0.02** |
| Bus idling (outside) | 20 | −0.10 | 0.20 | 0.64 | −0.14 | 0.14 | 0.32 | −0.93 | 0.35 | **0.01** | −0.19 | 0.11 | 0.08 | −0.31 | 0.18 | 0.09 |
| Exhaust (outside) | 8 | 0.62 | 0.30 | **0.04** | 0.30 | 0.21 | 0.15 | −0.06 | 0.55 | 0.92 | 0.34 | 0.17 | **0.04** | 0.06 | 0.27 | 0.82 |
| Teacher survey | | | | | | | | | | | | | | | | |
| How would you rate the general cleanliness of your classroom | No to constant (5 levels) | −0.10 | 0.12 | 0.42 | 0.00 | 0.09 | 0.99 | −0.41 | 0.23 | 0.08 | −0.17 | 0.07 | **0.02** | −0.02 | 0.12 | 0.90 |
| How often experienced carpet odors in classroom | No to constant (6 levels) | 0.03 | 0.11 | 0.78 | 0.08 | 0.08 | 0.33 | 0.13 | 0.22 | 0.55 | 0.12 | 0.07 | 0.07 | 0.01 | 0.11 | 0.95 |
| How often experienced raw gasoline in classroom | No to constant (6 levels) | −0.46 | 0.82 | 0.58 | 0.82 | 0.61 | 0.18 | 3.49 | 1.54 | **0.03** | −0.29 | 0.50 | 0.57 | 1.93 | 0.75 | **0.01** |
| How often experienced asphalt in classroom | No to constant (6 levels) | 0.68 | 0.41 | 0.10 | 0.85 | 0.30 | **0.01** | 1.39 | 0.80 | 0.08 | 0.10 | 0.26 | 0.70 | 1.17 | 0.38 | **0.00** |

Bold values are statistically significant ($p < 0.05$). [1] β: coefficients of linear regression models that are the slopes of models. [2] SE: standard error of the coefficients. [3] *p*: *p* -value for each model.

**Table S9.** Statistics of indoor VOC concentrations by grade in schools in the present study.

| **Indoor VOC** | **Grade Level *** | |
|---|---|---|
| | **ANOVA** | **K-W** |
| Aromatics | | |
| Benzene | 0.2273 | **0.0476** |
| Toluene | 0.8894 | 0.1565 |
| Ethylbenzene | 0.8943 | 0.5546 |
| *p*-Xylene, *m*-Xylene | 0.8981 | 0.6904 |
| *o*-Xylene | 0.9843 | 0.5654 |
| 1,2,4-TMB | 0.7562 | 0.5211 |
| BTEX | 0.8979 | 0.4641 |
| Alkanes | | |
| $C_6$ | 0.1456 | 0.1201 |
| $C_7$ | 0.9648 | 0.8331 |
| $C_{11}$ | 0.0567 | **0.0008** |
| $C_{12}$ | 0.8460 | 0.6092 |
| $C_{13}$ | 0.6591 | 0.7099 |
| $C_{14}$ | 0.9213 | 0.5847 |
| $C_{15}$ | 0.2331 | 0.3908 |
| $C_{16}$ | 0.6452 | 0.3075 |
| MCH | 0.6903 | 0.5876 |
| Alkanes | 0.3321 | 0.6263 |
| Terpenes | | |
| $\alpha$-Pinene | 0.1119 | 0.7426 |
| *d*-Limonene | 0.8395 | 0.9068 |
| Terpenes | 0.7100 | 0.8744 |
| Other | | |
| Ethyl acetate | 0.5407 | 0.3887 |
| MIBK | 0.8143 | 0.3650 |
| Naphthalene | 0.7970 | 0.8207 |
| DCM | 0.4223 | 0.8336 |
| Chloroform | 0.5415 | 0.7053 |
| *p*-DCB | 0.6760 | 0.6692 |
| Formaldehyde | 0.4149 | 0.2123 |
| TTVOCs | 0.7858 | 0.8451 |

* grade level = PK-K, 1–3.5, 4–8, others (art, music, special, and resources); *n* for grade PK-K = 22, 1–3.5 = 51, 4–8 = 60, others = 7; Bold values are statistically significant ($p < 0.05$).

**Table S10.** Statistics of VOC concentrations measured indoors and outdoors by season in schools in the present study.

| Parameter | Fall (μg/m³) | | | | Winter (μg/m³) | | | | Spring (μg/m³) | | | | Season: Fall, Winter, and Spring | | | |
|---|---|---|---|---|---|---|---|---|---|---|---|---|---|---|---|---|
| | Indoor ($n$ = 42) | | Outdoor ($n$ = 11) | | Indoor ($n$ = 50) | | Outdoor ($n$ = 11) | | Indoor ($n$ = 52) | | Outdoor ($n$ = 13) | | Indoor | | Outdoor | |
| Temp (°C) | 22.2± 1.5 | | 16.7 ± 3.4 | | 21.8 ± 1.4 | | 6.3 ± 5.9 | | 22.3 ± 1.8 | | 12.4 ± 4.4 | | | | | |
| RH (%) | 39.1 ± 8.9 | | 52.8 ± 11.7 | | 24.2 ± 7.3 | | 56.3 ± 10.1 | | 25.1 ± 7.2 | | 56.5 ± 14.5 | | | | | |
| Avg. AER ($h^{-1}$) | 0.8 ± 0.8 | | - | | 0.7 ± 0.4 | | - | | 0.8 ± 0.4 | | - | | | | | |
| Daytime AER ($h^{-1}$) | 1.4 ± 1.5 | | - | | 1.3 ± 0.8 | | - | | 1.5 ± 0.8 | | - | | | | | |
| VOC | Ave | Med | Ave | Med | Ave | Med | Ave | Med | Ave | Med | Ave | Med | ANOVA | K-W | ANOVA | K-W |
| Benzene | 0.67 | 0.43 | 0.43 | 0.31 | 0.52 | 0.43 | 0.63 | 0.51 | 0.36 | 0.35 | 0.56 | 0.54 | **0.01** | **0.01** | 0.70 | 0.42 |
| Toluene | 3.46 | 1.08 | 0.74 | 0.44 | 1.37 | 0.52 | 1.02 | 0.51 | 0.90 | 0.58 | 1.08 | 0.42 | 0.20 | **0.00** | 0.82 | 0.91 |
| Ethylbenzene | 0.40 | 0.19 | 0.19 | 0.07 | 0.23 | 0.07 | 0.10 | 0.07 | 0.09 | 0.07 | 0.07 | 0.07 | **0.00** | **0.00** | 0.12 | 0.41 |
| *p,m*-Xylene | 1.69 | 0.90 | 0.70 | 0.14 | 0.76 | 0.24 | 0.30 | 0.16 | 0.38 | 0.31 | 0.13 | 0.08 | **0.00** | **0.00** | 0.08 | **0.04** |
| *o*-Xylene | 0.53 | 0.33 | 0.28 | 0.05 | 0.24 | 0.05 | 0.10 | 0.05 | 0.15 | 0.05 | 0.06 | 0.05 | **0.00** | **0.00** | 0.07 | 0.06 |
| 1,2,4-TMB | 0.42 | 0.36 | 0.28 | 0.03 | 0.43 | 0.22 | 0.44 | 0.03 | 0.13 | 0.03 | 0.04 | 0.03 | **0.00** | **0.00** | 0.41 | 0.11 |
| BTEX | 6.75 | 3.54 | 2.33 | 1.14 | 3.12 | 1.42 | 2.15 | 1.99 | 1.88 | 1.55 | 1.90 | 1.44 | **0.01** | **0.00** | 0.82 | 0.98 |
| $C_6$ | 3.31 | 2.38 | 3.62 | 2.66 | 2.59 | 2.09 | 3.05 | 1.95 | 3.59 | 2.16 | 3.56 | 2.06 | 0.32 | 0.38 | 0.55 | 0.26 |
| $C_7$ | 1.16 | 0.19 | 0.18 | 0.12 | 1.09 | 0.12 | 0.20 | 0.12 | 0.53 | 0.12 | 0.29 | 0.12 | 0.10 | **0.05** | 0.77 | 1.00 |
| $C_{11}$ | 0.35 | 0.06 | 0.06 | 0.06 | 0.23 | 0.06 | 0.06 | 0.06 | 0.30 | 0.06 | 0.06 | 0.06 | 0.69 | 0.60 | 1.00 | 1.00 |
| $C_{12}$ | 0.39 | 0.22 | 0.22 | 0.22 | 0.55 | 0.22 | 0.23 | 0.22 | 0.73 | 0.55 | 0.25 | 0.22 | **0.00** | **0.00** | 0.60 | 0.81 |
| $C_{13}$ | 0.14 | 0.08 | 0.08 | 0.08 | 0.21 | 0.08 | 0.08 | 0.08 | 0.17 | 0.08 | 0.11 | 0.08 | 0.38 | 0.20 | 0.41 | 0.40 |
| $C_{14}$ | 1.57 | 1.15 | 0.07 | 0.07 | 0.98 | 0.80 | 0.11 | 0.07 | 1.37 | 1.07 | 0.07 | 0.07 | **0.05** | **0.03** | 0.15 | 0.15 |
| $C_{15}$ | 0.51 | 0.04 | 0.04 | 0.04 | 0.28 | 0.04 | 0.04 | 0.04 | 0.68 | 0.20 | 0.04 | 0.04 | 0.06 | **0.02** | 1.00 | 1.00 |
| $C_{16}$ | 0.23 | 0.06 | 0.06 | 0.06 | 0.24 | 0.06 | 0.06 | 0.06 | 0.44 | 0.06 | 0.06 | 0.06 | **0.05** | **0.01** | 1.00 | 1.00 |
| MCH | 2.81 | 1.25 | 1.25 | 1.25 | 4.48 | 1.25 | 1.89 | 1.25 | 1.81 | 1.25 | 3.03 | 1.25 | 0.08 | **0.02** | 0.15 | 0.23 |
| Alkanes | 7.66 | 5.52 | 4.34 | 3.76 | 6.16 | 5.21 | 3.83 | 3.36 | 7.81 | 6.55 | 4.43 | 4.54 | 0.15 | 0.21 | 0.51 | 0.45 |
| *α*-Pinene | 0.61 | 0.17 | 0.54 | 0.08 | 3.45 | 0.11 | 0.08 | 0.08 | 0.44 | 0.08 | 0.08 | 0.08 | **0.03** | 0.22 | 0.29 | 0.09 |
| *d*-Limonene | 15.09 | 4.56 | 0.18 | 0.13 | 15.11 | 4.36 | 0.13 | 0.13 | 4.60 | 2.60 | 0.13 | 0.13 | **0.03** | **0.01** | 0.31 | 0.31 |
| Terpenes | 15.7 | 4.73 | 0.72 | 0.21 | 18.56 | 4.44 | 0.21 | 0.21 | 5.05 | 2.81 | 0.21 | 0.21 | **0.02** | **0.01** | 0.22 | **0.02** |
| Ethyl acetate | 0.55 | 0.25 | 0.25 | 0.25 | 0.87 | 0.25 | 0.25 | 0.25 | 0.84 | 0.25 | 0.25 | 0.25 | 0.42 | 0.44 | 1.00 | 1.00 |
| MIBK | 0.17 | 0.04 | 0.04 | 0.04 | 0.26 | 0.04 | 0.04 | 0.04 | 0.20 | 0.04 | 0.04 | 0.04 | 0.64 | **0.03** | 1.00 | 1.00 |
| Naphthalene | 0.23 | 0.12 | 0.31 | 0.12 | 0.26 | 0.13 | 0.14 | 0.12 | 0.24 | 0.12 | 0.12 | 0.12 | 0.91 | 0.07 | 0.17 | 0.29 |
| DCM | 2.00 | 0.75 | 2.20 | 0.75 | 1.50 | 0.75 | 2.53 | 0.75 | 4.63 | 0.75 | 2.46 | 0.75 | **0.04** | 0.78 | 0.98 | 0.69 |
| Chloroform | 1.11 | 0.13 | 1.94 | 0.13 | 1.75 | 0.13 | 1.25 | 0.13 | 2.14 | 0.13 | 3.40 | 1.54 | 0.26 | 0.09 | 0.32 | 0.29 |
| *p*-DCB | 0.25 | 0.03 | 0.03 | 0.03 | 0.07 | 0.03 | 0.03 | 0.03 | 0.03 | 0.03 | 0.03 | 0.03 | **0.00** | **0.00** | 1.00 | 1.00 |
| Formaldehyde | 11.59 | 9.00 | - | - | 8.39 | 6.00 | - | - | 6.13 | 6.00 | - | - | **0.00** | **0.00** | - | - |
| TTVOCs | 49.33 | 36.61 | 10.92 | 8.96 | 45.89 | 32.49 | 10.12 | 10.40 | 30.89 | 24.71 | 12.76 | 9.81 | 0.07 | 0.22 | 0.37 | 0.71 |

Bold values are statistically significant ($p < 0.05$); RH: relative humidity; AER: air exchange rate.

**Table S11.** Statistics of VOC concentrations measured indoors and outdoors by dominant identified community outdoor source type in schools in the present study.

| **VOC** | **No source (μg/m³)** | | | | **Road (μg/m³)** | | | | **Industrial (μg/m³)** | | | | **Community: No Source, Road, and Industrial** | | | |
|---|---|---|---|---|---|---|---|---|---|---|---|---|---|---|---|---|
| | **Indoor ($n$ = 50)** | | **Outdoor ($n$ = 12)** | | **Indoor ($n$ = 43)** | | **Outdoor ($n$ = 11)** | | **Indoor ($n$ = 51)** | | **Outdoor ($n$ = 12)** | | **Indoor** | | **Outdoor** | |
| | **Ave** | **Med** | **Ave** | **Med** | **Ave** | **Med** | **Ave** | **Med** | **Ave** | **Med** | **Ave** | **Med** | **ANOVA** | **K-W** | **ANOVA** | **K-W** |
| Benzene | 0.46 | 0.39 | 0.45 | 0.47 | 0.40 | 0.39 | 0.46 | 0.43 | 0.65 | 0.47 | 0.71 | 0.64 | 0.06 | 0.07 | 0.29 | 0.44 |
| Toluene | 2.95 | 0.96 | 0.62 | 0.41 | 0.76 | 0.55 | 0.67 | 0.32 | 1.59 | 0.74 | 1.55 | 0.56 | 0.33 | **0.02** | 0.18 | 0.34 |
| Ethylbenzene | 0.38 | 0.11 | 0.15 | 0.07 | 0.11 | 0.07 | 0.10 | 0.07 | 0.19 | 0.07 | 0.10 | 0.07 | **0.00** | **0.00** | 0.59 | 0.86 |
| *p,m*-Xylene | 1.47 | 0.64 | 0.57 | 0.14 | 0.45 | 0.23 | 0.26 | 0.08 | 0.74 | 0.40 | 0.25 | 0.11 | **0.00** | **0.00** | 0.45 | 0.44 |
| *o*-Xylene | 0.50 | 0.28 | 0.20 | 0.05 | 0.14 | 0.05 | 0.13 | 0.05 | 0.22 | 0.05 | 0.09 | 0.05 | **0.00** | **0.00** | 0.60 | 0.63 |
| 1,2,4-TMB | 0.31 | 0.20 | 0.15 | 0.03 | 0.20 | 0.14 | 0.16 | 0.03 | 0.45 | 0.13 | 0.41 | 0.03 | 0.07 | 0.29 | 0.61 | 0.87 |
| BTEX | 5.75 | 2.63 | 2.00 | 1.20 | 1.86 | 1.30 | 1.61 | 0.96 | 3.39 | 1.84 | 2.70 | 0.34 | **0.05** | **0.00** | 0.50 | 0.28 |
| $C_6$ | 2.52 | 1.96 | 2.62 | 2.34 | 3.75 | 2.55 | 4.37 | 2.71 | 3.35 | 2.27 | 3.35 | 3.04 | 0.22 | **0.01** | 0.17 | 0.21 |
| $C_7$ | 0.96 | 0.12 | 0.18 | 0.12 | 0.31 | 0.12 | 0.12 | 0.12 | 1.43 | 0.12 | 0.37 | 0.12 | **0.01** | **0.01** | 0.31 | 0.38 |
| $C_{11}$ | 0.28 | 0.06 | 0.06 | 0.06 | 0.16 | 0.06 | 0.06 | 0.06 | 0.43 | 0.06 | 0.06 | 0.06 | 0.17 | 0.22 | 1.00 | 1.00 |
| $C_{12}$ | 0.57 | 0.39 | 0.22 | 0.22 | 0.62 | 0.22 | 0.22 | 0.22 | 0.54 | 0.22 | 0.26 | 0.22 | 0.65 | 0.54 | 0.23 | 0.16 |
| $C_{13}$ | 0.16 | 0.08 | 0.08 | 0.08 | 0.12 | 0.08 | 0.08 | 0.08 | 0.24 | 0.08 | 0.12 | 0.08 | 0.09 | **0.05** | 0.41 | 0.40 |
| $C_{14}$ | 1.25 | 1.00 | 0.07 | 0.07 | 1.53 | 1.25 | 0.07 | 0.07 | 1.18 | 0.84 | 0.10 | 0.07 | 0.28 | **0.01** | 0.15 | 0.15 |
| $C_{15}$ | 0.26 | 0.04 | 0.04 | 0.04 | 0.61 | 0.04 | 0.04 | 0.04 | 0.66 | 0.04 | 0.04 | 0.04 | 0.06 | **0.02** | 1.00 | 1.00 |
| $C_{16}$ | 0.24 | 0.06 | 0.06 | 0.06 | 0.38 | 0.06 | 0.06 | 0.06 | 0.33 | 0.06 | 0.06 | 0.06 | 0.40 | 0.66 | 1.00 | 1.00 |
| MCH | 2.82 | 1.25 | 1.25 | 1.25 | 1.60 | 1.25 | 1.25 | 1.25 | 4.55 | 1.25 | 3.76 | 1.25 | 0.07 | **0.01** | **0.01** | **0.01** |
| Alkanes | 6.24 | 5.27 | 3.33 | 2.99 | 7.48 | 6.76 | 5.02 | 3.36 | 8.16 | 6.52 | 4.36 | 4.21 | 0.18 | 0.29 | 0.19 | 0.18 |
| $\alpha$-Pinene | 0.33 | 0.09 | 0.09 | 0.08 | 0.61 | 0.08 | 0.53 | 0.08 | 3.70 | 0.08 | 0.08 | 0.08 | **0.02** | 0.57 | 0.32 | 0.56 |
| *d*-Limonene | 5.66 | 3.54 | 0.17 | 0.13 | 11.59 | 4.08 | 0.13 | 0.13 | 17.61 | 2.92 | 0.13 | 0.13 | **0.05** | 0.40 | 0.41 | 0.40 |
| Terpenes | 5.99 | 3.72 | 0.27 | 0.21 | 12.20 | 4.29 | 0.66 | 0.21 | 21.31 | 4.28 | 0.21 | 0.21 | **0.02** | 0.46 | 0.36 | 0.38 |
| Ethyl acetate | 0.95 | 0.25 | 0.25 | 0.25 | 0.53 | 0.25 | 0.25 | 0.25 | 0.83 | 0.25 | 0.25 | 0.25 | 0.28 | 0.21 | 1.00 | 1.00 |
| MIBK | 0.33 | 0.04 | 0.04 | 0.04 | 0.07 | 0.04 | 0.04 | 0.04 | 0.23 | 0.04 | 0.04 | 0.04 | **0.04** | 0.00 | 1.00 | 1.00 |
| Naphthalene | 0.21 | 0.12 | 0.17 | 0.12 | 0.22 | 0.12 | 0.26 | 0.12 | 0.31 | 0.12 | 0.12 | 0.12 | 0.28 | 0.95 | 0.46 | 0.79 |
| DCM | 3.70 | 0.75 | 1.33 | 0.75 | 1.85 | 0.75 | 2.25 | 0.75 | 2.76 | 0.75 | 3.61 | 0.75 | 0.40 | 0.71 | 0.30 | 0.33 |
| Chloroform | 0.78 | 0.13 | 0.80 | 0.13 | 3.05 | 1.66 | 4.29 | 2.90 | 1.41 | 0.13 | 1.88 | 0.13 | **0.00** | **0.00** | 0.07 | **0.05** |
| *p*-DCB | 0.12 | 0.03 | 0.03 | 0.03 | 0.20 | 0.14 | 0.16 | 0.03 | 0.45 | 0.13 | 0.03 | 0.03 | 0.45 | 0.20 | 1.00 | 1.00 |
| Formaldehyde | 9.22 | 6.00 | - | - | 8.45 | 6.00 | - | - | 8.15 | 6.00 | - | - | 0.49 | 0.14 | - | - |
| TTVOCs | 36.33 | 28.10 | 6.60 | 5.56 | 37.71 | 32.44 | 12.51 | 10.40 | 51.60 | 32.11 | 15.04 | 12.73 | **0.05** | 0.44 | **0.04** | **0.02** |

Bold values are statistically significant ($p < 0.05$).

**Table S12.** Comparison of indoor and outdoor VOC concentrations by building type in schools in the present study.

| VOC | Indoors (µg/m³) | | | | | | | | | | | Outdoors (µg/m³) | | | | | | | | | | |
|---|---|---|---|---|---|---|---|---|---|---|---|---|---|---|---|---|---|---|---|---|---|---|
| | Conventional (*n* = 39) | | | Energy Start (*n* = 58) | | | LEED (*n* = 47) | | | *p*-Value * | | Conventional (*n* = 10) | | | Energy Start (*n* = 15) | | | LEED (*n* = 10) | | | *p*-Value * | |
| | % DF | Ave | Med | % DF | Ave | Med | % DF | Ave | Med | ANOVA | KW-Test | % DF | Ave | Med | % DF | Ave | Med | % DF | Ave | Med | ANOVA | KW-Test |
| Benzene | 97 | 0.04 | 0.49 | 100 | 0.05 | 0.41 | 79 | 0.06 | 0.34 | **0.01** | **0.00** | 90 | 0.59 | 0.60 | 93 | 0.61 | 0.53 | 70 | 0.39 | 0.39 | 0.50 | 0.40 |
| Toluene | 100 | 2.21 | 0.73 | 100 | 2.85 | 0.88 | 100 | 1.18 | 0.59 | 0.27 | 0.14 | 100 | 0.76 | 0.49 | 100 | 0.84 | 0.33 | 90 | 1.34 | 1.17 | 0.70 | 0.28 |
| Ethylbenzene | 41 | 1.98 | 0.07 | 37 | 0.07 | 0.07 | 30 | 0.07 | 0.07 | **0.00** | 0.11 | 30 | 0.20 | 0.07 | 13 | 0.09 | 0.07 | 10 | 0.08 | 0.07 | 0.09 | 0.14 |
| *p,m*-Xylene | 74 | 0.39 | 0.49 | 84 | 0.24 | 0.48 | 81 | 0.10 | 0.30 | **0.00** | 0.12 | 40 | 0.71 | 0.08 | 53 | 0.25 | 0.11 | 40 | 0.18 | 0.08 | 0.10 | 0.62 |
| *o*-Xylene | 49 | 0.05 | 0.05 | 53 | 0.05 | 0.09 | 49 | 0.06 | 0.09 | **0.01** | 0.37 | 30 | 0.29 | 0.05 | 20 | 0.09 | 0.05 | 20 | 0.08 | 0.05 | 0.08 | 0.57 |
| 1,2,4-TMB | 77 | 0.08 | 0.24 | 60 | 0.06 | 0.18 | 42 | 0.24 | 0.03 | 0.12 | **0.00** | 40 | 0.30 | 0.03 | 13 | 0.28 | 0.03 | 40 | 0.13 | 0.03 | 0.74 | 0.28 |
| BTEX | 100 | 4.66 | 1.64 | 100 | 3.25 | 2.04 | 100 | 1.47 | 1.33 | 0.14 | **0.01** | 100 | 2.54 | 1.39 | 100 | 1.87 | 1.19 | 90 | 2.06 | 1.67 | 0.62 | 0.67 |
| $C_6$ | 100 | 1.07 | 2.31 | 94 | 2.60 | 2.32 | 93 | 2.78 | 1.85 | 0.20 | 0.12 | 100 | 4.30 | 3.86 | 93 | 2.50 | 2.35 | 90 | 3.92 | 2.51 | 0.17 | 0.12 |
| $C_7$ | 33 | 0.58 | 0.12 | 45 | 0.59 | 1.55 | 23 | 0.33 | 0.12 | 0.16 | 0.30 | 10 | 0.19 | 0.12 | 13 | 0.32 | 0.12 | 0 | <0.12 | <0.12 | 0.71 | 0.96 |
| $C_{11}$ | 23 | 14.54 | 0.06 | 24 | 6.38 | 0.06 | 2 | 15.48 | 0.06 | 0.96 | 0.37 | 0 | <0.06 | <0.06 | 0 | <0.06 | <0.06 | 0 | <0.06 | <0.06 | 1.00 | 1.00 |
| $C_{12}$ | 21 | 0.43 | 0.22 | 52 | 0.50 | 0.30 | 77 | 0.29 | 0.74 | **0.00** | **0.00** | 0 | <0.22 | <0.22 | 13 | 0.24 | 0.22 | 20 | 0.23 | 0.22 | 0.55 | 0.43 |
| $C_{13}$ | 3 | 0.23 | 0.08 | 21 | 0.15 | 0.08 | 23 | 0.32 | 0.08 | 0.13 | **0.03** | 0 | <0.08 | <0.08 | 7 | 0.11 | 0.08 | 0 | <0.08 | <0.08 | 0.49 | 0.48 |
| $C_{14}$ | 87 | 0.09 | 1.09 | 81 | 0.18 | 0.87 | 88 | 0.20 | 1.04 | **0.05** | 0.08 | 0 | <0.07 | <0.07 | 0 | <0.07 | <0.07 | 20 | 0.11 | 0.07 | 0.12 | 0.11 |
| $C_{15}$ | 31 | 1.61 | 0.04 | 23 | 0.96 | 0.04 | 58 | 1.46 | 0.44 | **0.01** | **0.00** | 0 | <0.04 | <0.04 | 0 | <0.04 | <0.04 | 0 | <0.04 | <0.04 | 1.00 | 1.00 |
| $C_{16}$ | 18 | 0.60 | 0.06 | 21 | 0.22 | 0.06 | 47 | 0.83 | 0.06 | 0.21 | **0.04** | 0 | <0.06 | <0.06 | 0 | <0.06 | <0.06 | 0 | <0.06 | <0.06 | 1.00 | 1.00 |
| MCH | 23 | 1.25 | 1.25 | 35 | 1.25 | 1.25 | 21 | 1.23 | 1.25 | 0.38 | 0.22 | 0 | <1.25 | <1.25 | 27 | 3.00 | 1.25 | 10 | 1.64 | 1.25 | 0.39 | 0.34 |
| Alkanes | 100 | 19.16 | 5.32 | 100 | 11.58 | 5.44 | 100 | 21.75 | 6.25 | 0.74 | 0.91 | 100 | 5.02 | 4.71 | 93 | 3.41 | 3.31 | 100 | 4.62 | 3.16 | 0.17 | 0.12 |
| α-Pinene | 49 | 0.46 | 0.08 | 48 | 0.30 | 0.08 | 44 | 0.17 | 0.08 | **0.01** | 0.34 | 20 | 0.58 | 0.08 | 0 | <0.08 | <0.08 | 0 | <0.08 | <0.08 | 0.25 | 0.06 |
| *d*-Limonene | 97 | 0.13 | 3.59 | 94 | 0.13 | 3.47 | 93 | 0.37 | 3.60 | 0.13 | 0.49 | 10 | 0.18 | 0.13 | 0 | <0.13 | <0.13 | 0 | <0.13 | <0.13 | 0.27 | 0.26 |
| Terpenes | 100 | 0.59 | 3.59 | 97 | 0.44 | 3.73 | 95 | 0.54 | 5.33 | 0.09 | 0.42 | 10 | 0.77 | 0.21 | 0 | <0.21 | <0.21 | 0 | <0.21 | <0.21 | 0.18 | **0.01** |
| Ethyl acetate | 23 | 2.51 | 0.25 | 19 | 0.96 | 0.25 | 37 | 2.33 | 0.25 | **0.03** | 0.18 | 0 | <0.25 | <0.25 | 0 | <0.25 | <0.25 | 0 | <0.25 | <0.25 | 1.00 | 1.00 |
| MIBK | 10 | 0.09 | 0.04 | 27 | 2.83 | 0.04 | 30 | 1.08 | 0.04 | 0.43 | 0.10 | 0 | <0.04 | <0.04 | 0 | <0.04 | <0.04 | 0 | <0.04 | <0.04 | 1.00 | 1.00 |
| Naphthalene | 49 | 0.27 | 0.12 | 31 | 0.44 | 0.12 | 44 | 0.85 | 0.12 | **0.05** | 0.07 | 30 | 0.34 | 0.12 | 7 | 0.12 | 0.12 | 0 | <0.12 | <0.12 | 0.09 | 0.06 |
| DCM | 21 | 1.59 | 0.75 | 27 | 3.07 | 0.75 | 9 | 3.43 | 0.75 | 0.42 | 0.15 | 20 | 2.25 | 0.75 | 40 | 3.29 | 0.75 | 20 | 1.22 | 0.75 | 0.84 | 0.81 |
| Chloroform | 41 | 0.16 | 0.13 | 13 | 0.13 | 0.13 | 49 | 0.16 | 0.13 | **0.03** | **0.00** | 50 | 3.08 | 1.51 | 7 | 0.40 | 0.13 | 70 | 4.26 | 2.86 | 0.07 | 0.01 |
| *p*-DCB | 13 | 0.41 | 0.03 | 18 | 0.33 | 0.03 | 12 | 0.21 | 0.03 | 0.09 | 0.37 | 0 | <0.03 | <0.03 | 0 | <0.03 | <0.03 | 0 | <0.03 | <0.03 | 1.00 | 1.00 |
| Formaldehyde | 31 | 10.23 | 6.00 | 32 | 9.03 | 6.00 | 5 | 6.34 | 6.00 | **0.00** | **0.00** | - | - | - | - | - | - | - | - | - | - | - |
| TTVOCs | 100 | 42.01 | 30.76 | 100 | 33.31 | 25.82 | 100 | 39.64 | 27.16 | 0.40 | 0.93 | 100 | 12.80 | 14.74 | 100 | 10.38 | 8.96 | 100 | 11.37 | 8.88 | 0.38 | 0.08 |

Bold values are statistically significant ($p < 0.05$). * *p*-value test for difference by building type.

**Table S13.** Comparison of indoor and outdoor VOC concentrations in school studies.

| Location | Year | Season | *n* | Indoor Concentration (μg/m³) | | | | | | | | *n* | Outdoor Concentration (μg/m³) | | | | | | | | Note | Reference |
|---|---|---|---|---|---|---|---|---|---|---|---|---|---|---|---|---|---|---|---|---|---|---|
| | | | | Benzene | Toluene | Ethyl Benzene | *m*/*p*-Xylene | *o*-Xylene | BTEX | *α*-Pinene | *d*-Limonene | | Benzene | Toluene | Ethyl Benzene | *m*/*p*-Xylene | *o*-Xylene | BTEX | *α*-Pinene | *d*-Limonene | | |
| Minneapolis, MN, USA | 2000 | Winter | 39 | 0.6 | 2.9 | 0.6 | 2.3 | 0.8 | 7.2 | 0.2 | 4.6 | 8 | 1.3 | 2.6 | 0.6 | 2.3 | 0.8 | 7.6 | 0.0 | 0.1 | Median | Adgate et al. [3] |
| | | Spring | 47 | 0.6 | 1.6 | 0.3 | 1.2 | 0.4 | 4.1 | 0.2 | 1.9 | 10 | 1.1 | 2.7 | 0.5 | 2 | 0.7 | 7.0 | 0.1 | 0.4 | | |
| California, USA | 2001 | fall | 4 | NA | 1.8 ** | NA | NA | NA | NA | NA | NA | 4 | NA | NA | NA | NA | NA | NA | NA | NA | | Hodgson et al. [4] |
| Ann Arbor, Michigan, USA | 2003 | Spring | 64 | 0.1 | 2.8 | 0.2 | 2.3 | 0.2 | 5.7 | 1.4 | 4.4 | 9 | 0.1 | 0.5 | <0.01 | 0 | <0.01 | 0.6 | 0.1 | 0.3 | Mean | Godwin and Batterman, [5] |
| Kocaeli, Turkey | 2006 | Summer | 3 | 7.5 | 55.1 | 11.1 | 9.6 | 5.9 | 89.1 | NA | NA | 3 | 4.8 | 18.2 | 6.1 | 6.8 | 5.6 | 41.4 | NA | NA | Mean | Pekey and Arslanbas [6] |
| | 2006–2007 | Winter | 3 | 19.8 | 77.8 | 12.4 | 21.5 | 11.2 | 142.6 | NA | NA | 3 | 16.4 | 42.0 | 8.8 | 20.2 | 10.0 | 97.4 | NA | NA | Mean | |
| 11 cities in Europe * | 2003–2008 | Winter | 188 | 4.4 | 12.6 | 2.4 | 6.2 | 2.2 | 27.8 | 3.2 | 9.4 | 188 | 3.2 | 11.5 | 2.2 | 5.7 | 2.0 | 24.6 | 0.1 | 0.3 | Mean | Geiss et al. [8] |
| | | Summer | 188 | 2.6 | 7.1 | 1.3 | 2.9 | 1.2 | 15.1 | 1.5 | 2.6 | 188 | 2.1 | 4.8 | 1.1 | 2.5 | 0.9 | 11.4 | 0.0 | 0.2 | Median | |
| Lisbon, Portugal | 2009 | Spring | 28 | 0.3 | 3.0 | 2.0 | 4.3 | 3.2 | 12.9 | NA | NA | 14 | 0.3 | 2.1 | 0.7 | 1.4 | 0.5 | 4.9 | NA | NA | Mean | Pegas et al. [9] |
| | | Autumn | 28 | 0.5 | 5.0 | 1.1 | 2.7 | 3.8 | 12.3 | NA | NA | 14 | 0.5 | 1.9 | 0.4 | 1.0 | 0.4 | 4.1 | NA | NA | | |
| | | Winter | 28 | 1.2 | 6.0 | 4.5 | 12.0 | 13.2 | 34.4 | NA | NA | 14 | 1.2 | 2.9 | 0.5 | 0.9 | 0.4 | 5.2 | NA | NA | | |
| Bari, Italy | NA | NA | 24 | 0.9 | 2.3 | 0.6 | 2.7 | NA | 6.4 | 4.9 | 6.7 | 8 | 0.6 | 1.9 | 0.5 | 1.1 | NA | 4.1 | 1.0 | 7.1 | Mean | de Gennaro et al. [12] |
| MI, IL, IN, OH, USA | 2015–2016 | Autumn | 42 | 0.4 | 1.1 | 0.2 | 0.9 | 0.3 | 3.5 | 0.2 | 4.6 | 11 | 0.3 | 0.4 | 0.1 | 0.1 | 0.1 | 1.1 | 0.1 | 0.1 | Median | This study |
| | | Winter | 50 | 0.4 | 0.5 | 0.1 | 0.2 | 0.1 | 1.4 | 0.1 | 4.4 | 11 | 0.5 | 0.5 | 0.1 | 0.2 | 0.1 | 2.0 | 0.1 | 0.1 | Median | |
| | | Spring | 52 | 0.4 | 0.6 | 0.1 | 0.3 | 0.1 | 1.6 | 0.1 | 2.6 | 13 | 0.5 | 0.4 | 0.1 | 0.1 | 0.1 | 1.4 | 0.1 | 0.1 | Median | |

* Arnhem (Netherlands), Nijmegen (Netherlands), Athens (Greece), Brussels (Belgium), Budapest (Hungary), Catania (Italy), Dublin (Ireland), Helsinki (Finland), Leipzig (Germany), Nicosia (Cyprus), Thessaloniki (Greece). 188 samples included public buildings, schools, and homes. ** Indoor minus outdoor concentration.

## One-Zone IAQ Model

A simple IAQ model was used for examination of the relationship between the AER and VOC concentration.

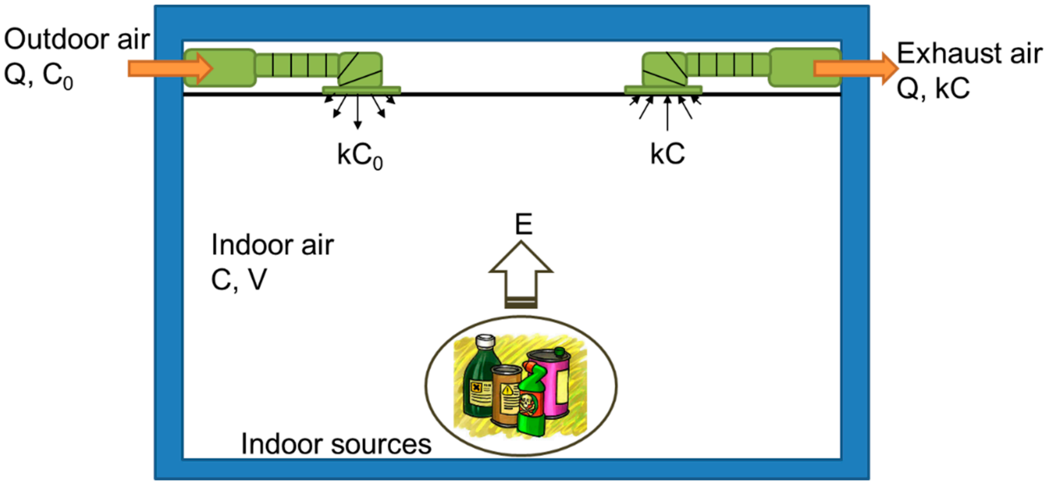


**Figure S1.** One-zone IAQ model.

The mass balance of contamination inside the room with is

$$V\frac{dC}{dt} = E + kQC_0 - kQC \quad (1)$$

At steady state, the indoor VOC concentration can be expressed as Equation (2) when the initial VOC concentration inside the room is negligible.

$$C = \left(\frac{E}{kQ} + C_0\right)\left(1 - e^{-knt}\right) \approx \frac{E}{kQ} + C_0 = \frac{E}{knV} + C_0 \quad (2)$$

Assuming there is no indoor source, Equation (2) can be simplified to:

$$C = C_0 \quad (3)$$

Assuming the outdoor air is very clean, Equation (2) can be simplified to:

$$C = \frac{E}{kQ} = \frac{E}{knV} \quad (4)$$

where $V$ = room volume (m$^3$); $C$ = room air VOC concentration (μg/m$^3$); $C_0$ = outdoor air VOC concentration (μg/m$^3$); $E$ = emission rate of indoor sources (μg/h); $n$ = air exchange rate (h$^{-1}$); and $k$ = a mixing factor.

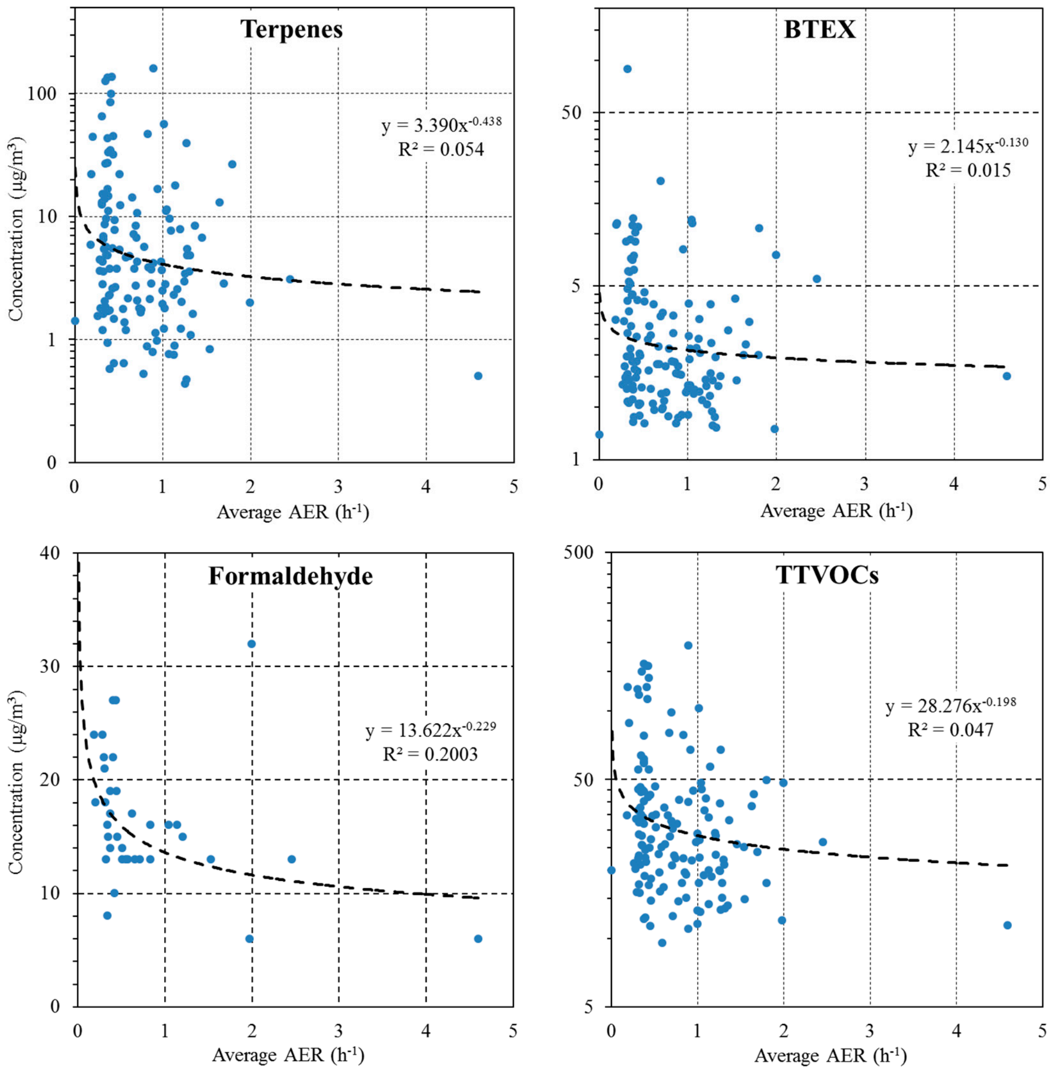


**Figure S2**. Indoor measured concentrations of terpenes, BTEX, formaldehyde, and TTVOCs against average AERs.

## References

1. Norback, D. Subjective indoor air quality in schools–The influence of high room temperature, carpeting, fleecy wall materials and volatile organic compounds (VOC). *Indoor Air* **1995**, *5*, 237–246.
2. Shendell, D.G.; Winer, A.M.; Stock, T.H.; Zhang, L.; Zhang, J.J.; Maberti, S.; Colome, S.D. Air concentrations of VOCs in portable and traditional classrooms: Results of a pilot study in Los Angeles county. *J. Expos. Anal. Environ. Epidemiol.* **2004**, *14*, 44–59.
3. Adgate, J.L.; Church, T.R.; Ryan, A.D.; Ramachandran, G.; Fredrickson, A.L.; Stock, T.H.; Morandi, M.T.; Sexton, K. Outdoor, indoor, and personal exposure to VOCs in children. *Environ. Health Perspect.* **2004**, *112*, 1386–1392.
4. Hodgson, A.T.; Shendell, D.G.; Fisk, W.J.; Apte, M.G. Comparison of predicted and derived measures of volatile organic compounds inside four new relocatable classrooms. *Indoor Air* **2004**, *14*, 135–144.
5. Godwin, C.; Batterman, S. Indoor air quality in Michigan schools. *Indoor Air* **2007**, *17*, 109–121.
6. Pekey, H.; Arslanbaş, D. The relationship between indoor, outdoor and personal VOC concentrations in homes, offices and schools in the metropolitan region of Kocaeli, Turkey. *Water Air Soil Poll.* **2008**, *191*, 113–129.
7. Fraga, S.; Ramos, E.; Martins, A.; Samúdio, M.J.; Silva, G.; Guedes, J.; Fernandes, E.O.; Barros, H. Indoor air quality and respiratory symptoms inporto schools. *Revista Portuguesa de Pneumologia* **2008**, *14*, 487–507.
8. Geiss, O.; Giannopoulos, G.; Tirendi, S.; Barrero-Moreno, J.; Larsen, B.R.; Kotzias, D. The airmex study—VOC measurements in public buildings and schools/kindergartens in eleven European cities: Statistical analysis of the data. *Atmos. Environ.* **2011**, *45*, 3676–3684.

9. Pegas, P.N.; Alves, C.A.; Evtyugina, M.G.; Nunes, T.; Cerqueira, M.; Franchi, M.; Pio, C.A.; Almeida, S.M.; Verde, S.C.; Freitas, M.C. Seasonal evaluation of outdoor/indoor air quality in primary schools in Lisbon. *J. Environ. Monitor.* **2011**, *13*, 657–667.
10. Pegas, P.N.; Alves, C.A.; Evtyugina, M.G.; Nunes, T.; Cerqueira, M.; Franchi, M.; Pio, C.A.; Almeida, S.M.; Freitas, M.C. Indoor air quality in elementary schools of Lisbon in spring. *Environ. Geochem. Health* **2011**, *33*, 455–468.
11. Banerjee, S.; Annesi-Maesano, I. Spatial variability of indoor air pollutants in schools. A multilevel approach. *Atmos. Environ.* **2012**, *61*, 558–561.
12. De Gennaro, G.; Farella, G.; Marzocca, A.; Mazzone, A.; Tutino, M. Indoor and outdoor monitoring of volatile organic compounds in school buildings: Indicators based on health risk assessment to single out critical issues. *Intern. J. Environ. Res. Public Health* **2013**, *10*, 6273–6291.
13. Chatzidiakou, L.; Mumovic, D.; Summerfield, A.J.; Hong, S.M.; Altamirano-Medina, H. A victorian school and a low carbon designed school: Comparison of indoor air quality, energy performance, and student health. *Indoor Built Environ.* **2014**, *23*, 417–432.
14. Madureira, J.; Paciencia, I.; Ramos, E.; Barros, H.; Pereira, C.; Teixeira, J.P.; Fernandes Ede, O. Children's health and indoor air quality in primary schools and homes in portugal-study design. *J. Toxicol. Environ. Health* **2015**, *78*, 915–930.

© 2017 by the authors; licensee MDPI, Basel, Switzerland. This article is an open access article distributed under the terms and conditions of the Creative Commons by Attribution (CC-BY) license (http://creativecommons.org/licenses/by/4.0/).
